# Live-cell analysis of IMPDH protein levels during yeast colony growth provides insights into the regulation of GTP synthesis

**DOI:** 10.1128/mbio.01021-24

**Published:** 2024-06-28

**Authors:** Erica L. Shand, Kieran Sweeney, Kaitlin E. Sundling, Megan N. McClean, David A. Brow

**Affiliations:** 1 Department of Biomolecular Chemistry, University of Wisconsin School of Medicine and Public Health, Madison, Wisconsin, USA; 2 Department of Biomedical Engineering, University of Wisconsin-Madison, Madison, Wisconsin, USA; 3 University of Wisconsin Carbone Cancer Center, University of Wisconsin School of Medicine and Public Health, Madison, Wisconsin, USA; Harvard Medical School, Boston, Massachusetts, USA

**Keywords:** transcription attenuation, nucleotide metabolism, microfluidics, mycophenolic acid, GTP biosynthesis

## Abstract

The purine nucleotides ATP and GTP are made from the common precursor inosine monophosphate (IMP). Maintaining the correct balance of these nucleotides for optimal cell growth is controlled in part by the enzyme IMP dehydrogenase (IMPDH), which catalyzes the first dedicated step of GTP biosynthesis. The regulation of IMPDH mRNA and protein levels in the yeast *S. cerevisiae* grown in liquid culture has been studied in some detail, but regulation of IMPDH protein under conditions of cellular crowding on a solid substrate has not been examined. Here, we report real-time, live-cell analysis of the accumulation of the Imd2 isoform of IMPDH in yeast cells forming a monolayer colony in a microfluidic device over a 50-hour time course. We observe two distinct phases of increased Imd2 accumulation: a guanine-insensitive phase early in outgrowth and a guanine-sensitive phase later, when cells become crowded. We show that the IMPDH inhibitor mycophenolic acid enhances both phases of increase. Deletion of a transcription attenuator upstream of the mRNA start site that decreases Imd2 mRNA synthesis in the presence of high GTP increases the baseline level of Imd2 protein 10-fold and abolishes guanine-sensitive but not guanine-insensitive induction. Our results suggest that at least two mechanisms of yeast Imd2 regulation exist, the known GTP-dependent attenuation of RNA polymerase II elongation and a GTP concentration-independent pathway that may be controlled by cell growth state. Live-cell analysis of IMPDH protein levels in a growing yeast colony confirms a known mechanism of regulation and provides evidence for an additional mode of regulation.

**IMPORTANCE:**

This study used live-cell microscopy to track changes in the level of a key enzyme in GTP nucleotide biosynthesis, inosine monophosphate dehydrogenase (IMPDH), during growth of a brewers yeast colony over 2 days in a microfluidic device. The results show that feedback regulation via transcription attenuation allows cells to adapt to nutrient limitation in the crowded environs of a yeast colony. They also identify a novel mode of regulation of IMPDH level that is not driven by guanine nucleotide availability.

## INTRODUCTION

**Fig 1:**
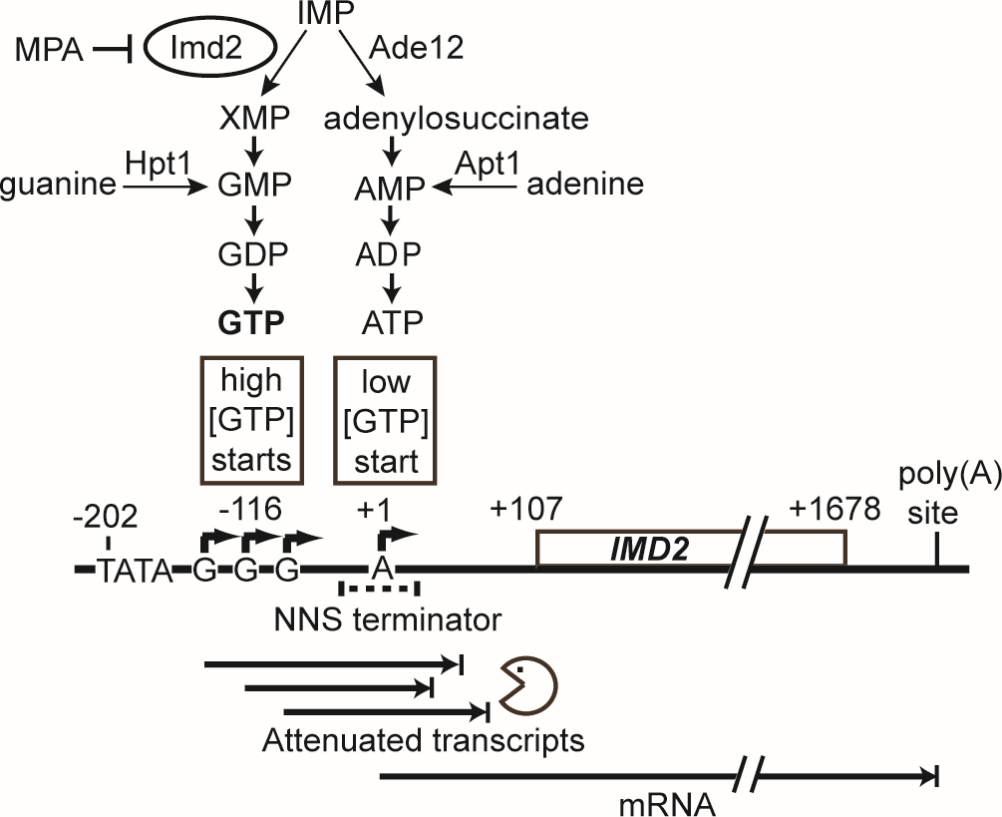
*IMD2* mRNA synthesis is regulated by GTP-dependent start site selection and transcriptional attenuation. Imd2 catalyzes the first committed step of GTP synthesis from IMP, which is also a precursor of ATP. *IMD2* has multiple transcription start sites (TSS, bent arrows). Upstream TSS are used when cellular GTP is sufficient, producing transcripts containing a Nrd1-Nab3-Sen1 (NNS) terminator that terminate prematurely and are degraded by the nuclear exosome. When cellular GTP is deficient, a downstream TSS is preferentially used, resulting in full-length mRNA [adapted from reference (8)].

The purine nucleotide guanylate is an essential cellular metabolite used for RNA and DNA synthesis, mRNA translation, signaling, and other biological processes. GMP and AMP are synthesized from the common metabolite inosine monophosphate (IMP), with the first step catalyzed by IMP dehydrogenase (IMPDH) or adenylosuccinate synthase, respectively (Fig. 1). Adenine nucleotides are more abundant than guanine nucleotides in yeast and human cells (1, 2), and maintenance of the optimal balance of purine nucleotides is, in part, regulated by IMPDH levels. High levels of IMPDH expression are associated with increased cell proliferation and malignancy in mammalian cells (3), and inhibiting IMPDH catalytic activity has been suggested as a potential chemotherapy (4–7).

In the yeast *Saccharomyces cerevisiae*, IMPDH is produced from three genes, *IMD2-4*, with *IMD1* producing no functional transcript (9, 10). When GTP is abundant, *IMD3* and *IMD4* contribute most of the cell’s IMPDH. However, when GTP is depleted, for example, by treatment with the specific IMPDH inhibitor mycophenolic acid (MPA; 10), transcription of all three genes is induced (11, 12). Only Imd2 exhibits significant resistance to MPA inhibition and is thus required for yeast cells to survive MPA treatment (10, 13).

Regulation of *IMD2* transcription has been characterized in suspension yeast cell culture. When intracellular GTP levels are high, RNA polymerase II (RNAP II) scanning downstream from the *IMD2* TATA box predominantly starts transcription at sites encoding one to three initial guanylate residues (Fig. 1) (8, 14, 15). Transcripts starting at these “G” sites incorporate a sequence that is recognized by the NNS transcription termination pathway (16), which signals 3′-end formation and degradation of the nascent transcript by the nuclear exosome. Thus, little Imd2 mRNA is produced. When intracellular GTP levels are low, RNAP II scans through the G start sites, allowing it to reach a downstream adenylate start site that produces functional Imd2 mRNA. In human and hamster cells, IMPDH mRNA has been shown to be regulated by a nuclear mechanism that is possibly analogous to the one described in yeast (17). However, additional mechanisms of regulation of Imd2 synthesis, both transcriptional and posttranscriptional, may exist, especially given evidence that all yeast Imd proteins can be crosslinked to polyadenylated RNA (18).

Prior studies on the regulation of Imd2 expression focused primarily on mRNA synthesis rather than protein synthesis. Furthermore, these studies were done with cells in suspension culture and measured population-wide changes in gene expression. Recent studies have demonstrated that expression of some metabolic enzymes in microbes is regulated by the location of individual cells within a growing colony (19, 20). Furthermore, cells within a colony may differentiate into metabolite-producing and metabolite-consuming cells during nutrient limitation (21, 22). These results show that a yeast colony is a complex microenvironment with variability between cells possibly driven by access to nutrients, as may also occur in human tissues. Thus, we sought to investigate Imd2 regulation in individual cells under conditions that mimic the cell crowding and variable access to nutrients present in a yeast colony growing in nature.

Here, we tracked GFP-tagged Imd2 abundance using time-lapse microscopy of growing yeast cells in a constant-flow microfluidic device that constrains the cells to a monolayer. This approach enabled us to monitor Imd2 accumulation in real time as cells formed colonies with constant perfusion of nutrients. We observed two distinct induction phases of Imd2-GFP during cell growth with or without MPA addition. The first phase was refractory to the presence of supplemental guanine, while the second phase was abrogated by supplemental guanine. Interestingly, MPA treatment amplified both phases. The insensitivity of the first phase of Imd2 induction to guanine supplementation suggests it does not involve GTP-dependent changes in start site selection by RNAP II. Moreover, deletion of the attenuator region increased basal expression of Imd2-GFP about 10-fold and eliminated guanine-sensitive Imd2-GFP induction but did not abolish guanine-insensitive induction. Our results indicate that restricted access to nutrients due to physical crowding induces Imd2 expression in the absence of supplemental guanine and that another mechanism for induction of Imd2 expression regardless of guanine supplementation is operative in this experimental system of colony growth.

## RESULTS

### Imd2-GFP expression during yeast cell growth can be monitored in a constant flow microfluidic device for 50 hours

Prior studies identified feedback regulation of *IMD2* gene transcription based on the influence of intracellular GTP concentration on start site selection (8, 14; Fig. 1). We sought to determine if this is the major regulatory mechanism for Imd2 protein expression or if other, possibly posttranscriptional mechanisms also exist. Furthermore, we sought to monitor Imd2 protein levels in dividing cells over time to assess the kinetics of changes due to the growth state or the addition of compounds that increase or decrease intracellular GTP levels. Measurement of protein rather than mRNA levels monitors the combined effects of all steps of gene expression as well as protein turnover.

**Fig 2:**
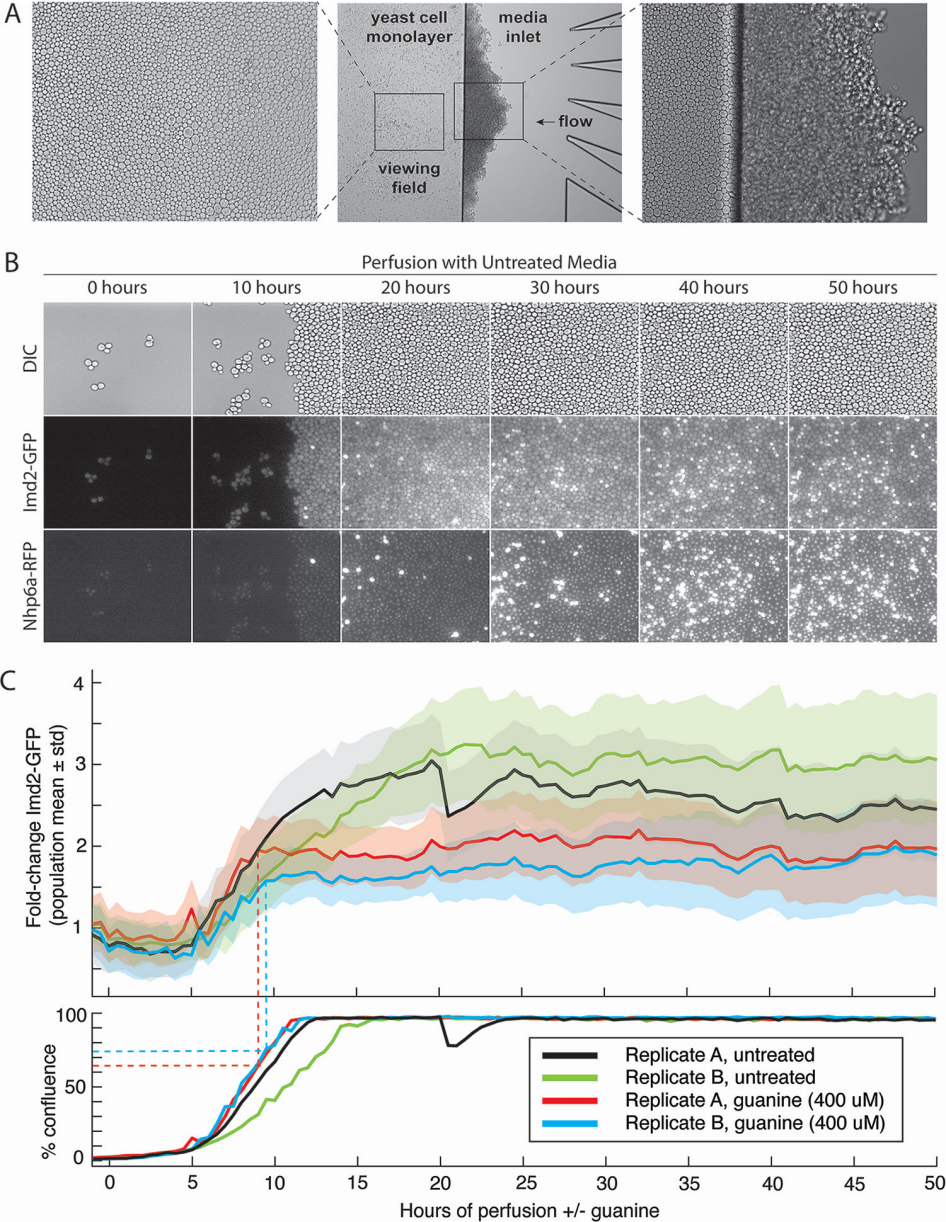
Single-cell measurement of Imd2-GFP protein accumulation over 50 hours with and without guanine supplementation. (**A**) Differential interference contrast (DIC) images of a representative viewing field after 50 hours of perfusion. Left panel: viewing field imaged with a 40× objective. Center panel: image with a 10× objective of the edge of the yeast cell monolayer adjacent to the media inlet channels. Cells that were pushed into the inlet by growth of the monolayer have formed a three-dimensional colony. Right panel: 40× objective image of yeast cells entering the media inlet from the monolayer. (**B**) DIC, Imd2-GFP, or Nhp6a-TagRFP images of cells after 0, 10, 20, 30, 40, or 50 hours of perfusion with synthetic complete media with no additions. Approximately one-quarter of the standard viewing field is shown. Very bright cells in the RFP channel are likely dead (see main text). (**C**) Single-cell measurement of Imd2-GFP protein accumulation over 50 hours with and without guanine supplementation at 0 hours. Top panel: Imd2-GFP fold-change versus hours of perfusion with untreated media (black and green lines) or media supplemented with 400 µM guanine (red and blue lines). Both treatments were performed in biological replicate. The solid lines indicate population mean per cell GFP fluorescence, and shading indicates one standard deviation above and below the mean. Dotted lines indicate the time at which guanine supplementation stops the increase in Imd2-GFP levels and the percent confluence of the cells at that time. Bottom panel: the percentage of the viewing field occupied by cells over time.

To this end, we used the CellASIC microfluidic system that allows microscopic observation of live yeast cells constrained to a monolayer (Fig. 2A). The apparatus allows constant perfusion with fresh media of several different compositions for the duration of the experiment, in each of four simultaneous experiments. To quantify the response of Imd2 protein levels to changing growth conditions in live cells, we used a yeast strain, KES002, that produces an Imd2-GFP C-terminal fusion protein from the genomic *IMD2* locus. The strain also contains a chromosomal *NHP6A-TagRFP-T* gene fusion that can be used to visualize nuclei and identify dead cells (see below) (23). While we cannot rule out that the GFP-coding region in the DNA and RNA, or the GFP residues attached to Imd2, alters the regulation of Imd2 levels, the fact that Imd2-GFP cells survive MPA challenge (see below) indicates that Imd2-GFP is functional, because cells with *IMD2* deleted do not grow in the presence of 7 µg/mL MPA (10).

DIC and fluorescent images of each of four viewing fields were acquired every 30 minutes for 50 hours unless otherwise noted (Fig. 2B and C). A MATLAB pipeline was used to quantify the Imd2-GFP intensity in each cell at each time point (see Materials and Methods and Fig. S1). We optimized the starting number of cells by loading different cell concentrations, with optical densities at 600 nm (OD_600_) from 0.2 to 1.6. A cell density of 0.4 OD_600_ typically resulted in 12–20 cells initially retained in the viewing field and was used for all experiments. At 30°C in synthetic complete medium with no additions, cells usually achieved confluence after 10–15 hours of perfusion and continued to divide, resulting in spillover of cells from the monolayer into the area containing the media inlets (Fig. 2A). Cells tend to accumulate at the constriction separating the monolayer from the nutrient inlet during loading and, as they propagate, often enter the viewing field from the right (see Fig. 2B at 10 hours).

Prior to confluence, the Nhp6a-RFP fluorescence was relatively dim and localized to the nucleus, as expected. After confluence, an increasing number of cells exhibited strong RFP fluorescence that was diffuse throughout the cell (Fig. 2B; Video S1). The RFP-bright cells typically appeared first on the left side of the viewing field, furthest from the media inlet, and appeared small and irregular in shape in the DIC channel. These cells may experience decreased nutrients compared with those closer to the media inlet. We infer that the RFP-bright cells are dead or dying (24). In this experiment, many of the RFP-bright cells also appear bright in the GFP channel but in experiments involving MPA addition, this was not usually the case. In fact, RFP-bright cells were often GFP dark and irregularly shaped, consistent with being dead (see Fig. S4).

It was unclear whether nutrients reach cells deep within a confluent monolayer. To visualize the flow of small molecules through the confluent cells, we perfused the DNA-binding dye DAPI into confluent cells after 50 hours of growth. Within 10 minutes, cells on the right side of the viewing field, closest to the nutrient inlet channels, began to stain with DAPI (see Video S4). Cells furthest from the nutrient inlets (left side of the viewing field) took up to an hour to stain brightly with DAPI. We noted that a subset of cells in the viewing field exhibited much brighter DAPI staining than other cells and these cells also had a much brighter nuclear Nhp6a-RFP fluorescence (cf. Fig. S2B and C), consistent with these cells being dead or dying and more permeable to DAPI staining. These results indicate that, when cells are confluent, those on the inlet (right) side of the viewing field have access to perfused molecules minutes before those on the left side. Decreased access to nutrients could explain the greater number of apparently dead cells in the distal (left) half of the viewing field.

### Imd2-GFP expression is induced during yeast cell monolayer growth

To characterize the influence of cell growth state on Imd2-GFP expression, we observed cells in the microfluidic chamber for 50 hours of perfusion with synthetic complete (SC) medium and quantified Imd2-GFP expression relative to the initial Imd2-GFP level at time zero (Fig. 2C, black and green lines; Video S1). For the first 5 hours, the average per cell Imd2-GFP expression decreased slightly and then returned to its initial level. Over the next 15 hours, Imd2-GFP levels increased approximately threefold, after which they remained relatively constant for the remainder of the 50-hour experiment. The start of Imd2-GFP induction correlated with the start of a rapid increase in the area of the viewing field occupied by cells, and the end of induction occurred about 10 hours after complete coverage of the viewing field by cells (Fig. 2C).

Evidence that the elevated level of Imd2-GFP is maintained by high cell density came from an incident unique to this experiment. Between 20 and 20.5 hours of perfusion, replicate A of untreated cells experienced a disruption in the confluent cell mass that opened a channel through which medium could flow (Fig. S3, see Video S5). This disruption appeared as a sharp dip in the percent confluency plot and, interestingly, a closely coincident decrease in the per-cell average Imd2-GFP fluorescence (Fig. 2C). Inspection of the first micrograph taken after the channel formed, at 20.5 hours, revealed that many of the cells bordering the open channel were among the lowest 20th percentile for Imd2-GFP fluorescence in the viewing field (Fig. S3B). This observation suggests that the higher steady-state level of Imd2-GFP expression after cells reach maximum density is reversible if cells gain more access to nutrients. This finding is consistent with the induction of Imd2-GFP at high cell density resulting from nutrient restriction, potentially via decreased GTP levels and a consequent increase in Imd2 mRNA synthesis (Fig. 1).

### Imd2-GFP induction occurs in two phases: guanine independent and guanine sensitive

To test our hypothesis that decreased intracellular GTP drives increased Imd2 expression as cells approach confluence, we determined the effect of perfusing cells with SC medium supplemented with 400 µM guanine throughout the 50-hour time course. This concentration was based on a titration experiment (see Fig. 4A). Guanine should enter cells via the purine nucleobase transporter Fcy2 (25) and be converted to GMP by Hpt1 (26, 27), thus increasing the GTP level without requiring Imd2 catalytic activity (Fig. 1). Both guanine-treated and untreated cells exhibit a similar 1.5- to 2-fold increase in Imd2-GFP by 8 to 9 hours after the start of perfusion (Fig. 2C; Video S2), but as the cells approach confluence, guanine-treated cells plateau at a lower fold induction of Imd2-GFP (approximately twofold) compared with untreated cells (approximately threefold). Thus, Imd2-GFP protein induction appears to occur in two phases: the first phase occurs from 5 to 9 hours of perfusion, before cells reach high density, and is not diminished by exogenous guanine while the second phase occurs as cells reach maximum density and is abolished by guanine supplementation.

The ability of guanine supplementation to decrease the second phase of Imd2-GFP induction supports the model that intracellular GTP becomes limiting when cells are crowded and that low GTP levels induce increased synthesis of Imd2-GFP mRNA via altered RNAP II start site selection. Conversely, the fact that guanine supplementation did not affect the first phase of Imd2-GFP induction suggests that it occurs by some other mechanism.

### Mycophenolic acid strongly stimulates both phases of Imd2-GFP induction

To further investigate the guanine-dependence of Imd2 induction, we sought to decrease GTP levels using the drug MPA. MPA is a specific inhibitor of IMPDH catalytic activity (28) and reduces the steady-state level of intracellular GTP (29). MPA treatment strongly increases *IMD2* expression (11, 12) at least in part due to a shift in transcription initiation to *IMD2’s* downstream productive start site (Fig. 1; 8, 14).

**Fig 3:**
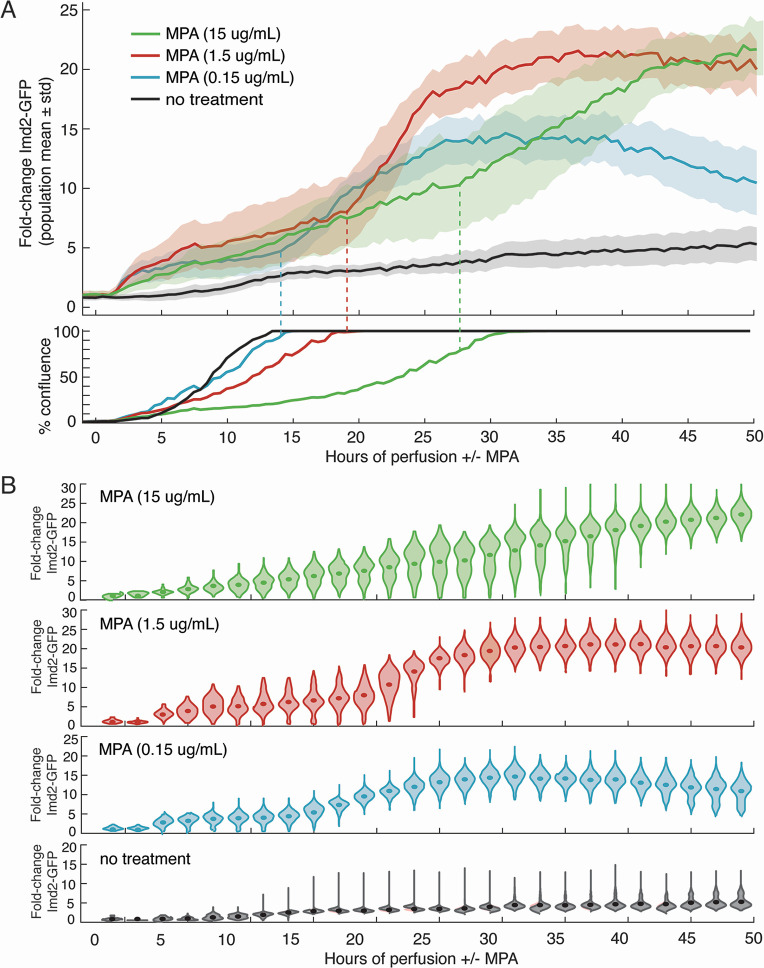
Dose-dependent induction of Imd2-GFP by MPA. (**A**) Top panel: population mean fold change in Imd2-GFP (solid lines) with one standard deviation above and below the mean (shaded areas) for cells treated with no MPA (black), 0.15 µg/mL (blue), 1.5 µg/mL (red), or 15 µg/mL MPA (green). Bottom panel: percentage of the viewing field occupied by cells over time. (**B**) Violin plots showing the distribution of Imd2-GFP expression across the cell population. Solid dots represent the population mean Imd2-GFP.

To determine if MPA affects one or both phases of Imd2-GFP induction and their sensitivity to guanine, we tested a range of concentrations. MPA concentrations used on yeast in published studies range from 0.03 µg/mL (29) to 100 µg/mL (30). To determine the concentration of MPA that induces maximum Imd2-GFP expression with minimal effects on growth rate, we treated yeast with successive 10-fold dilutions of MPA in SC medium starting at 15 µg/mL (47 µM) MPA. The highest MPA concentration has a strong inhibitory effect on cell growth and Imd2-GFP induction (Fig. 3A), most likely due to severe guanine nucleotide starvation. In contrast, 1.5 µg/mL MPA results in the highest level of Imd2-GFP induction with only a modest decrease in growth rate. A further 10-fold dilution of MPA to 0.15 µg/mL shows growth similar to untreated cells but results in a lower maximal induction of Imd2-GFP expression. Therefore, we chose 1.5 µg/mL (4.7 µM) MPA for all subsequent experiments.

Both the 0.15 and 1.5 µg/mL MPA-treated cells exhibited pronounced biphasic induction of Imd2-GFP (Fig. 3A). Phase 1 began about 1.5 hours after MPA treatment started and had a similar initial rate for these MPA concentrations, but the amplitude was smaller for the lower concentration. Phase 2 started earlier for 0.15 µg/mL MPA but at both concentrations was roughly coincident with achieving maximum confluence. In this regard, Phase 2 of MPA induction is similar to the guanine-sensitive phase of induction in the absence of MPA, while Phase 1 started much earlier in the presence of MPA than in its absence.

To better visualize the heterogeneity in cells’ response to MPA, we used violin plots to view the range of cellular Imd2-GFP induction (Fig. 3B). This analysis revealed the largest heterogeneity in cells treated with 15 µg/mL MPA. For example, at 35 hours, the level of Imd2- GFP ranged from no induction to 30-fold induction. At 1.5 µg/mL MPA, cells exhibited the largest heterogeneity at the transition from the first to second phase of induction, around 20 hours, but the time points after 40 hours look very similar to the 15 µg/mL MPA population. The 0.15 µg/mL MPA population exhibited the most coordinated induction in response to MPA.

A small number of MPA-untreated cells with bright GFP signal accumulate over time, starting around 10 hours (Fig. 3B, bottom) and may be senescent cells. Very bright cells in the RFP channel appear to be dead, as judged by their small volume and irregular shape in the DIC channel (Video S3; Fig. S4). These cells are usually dark in the GFP channel. Unexpectedly, the number of RFP-bright cells decreases with increasing MPA concentration (Fig. S5). We hypothesize that the slower growth rate at higher MPA concentrations reduced the total number of cell divisions and decreased the accumulation of dead cells. It is important to note that most confluent cells continue to divide and exit the viewing area (Video S3).

We tested the reproducibility of induction of Imd2-GFP by 1.5 µg/mL MPA by running four biological replicates simultaneously on the same microfluidic plate (Fig. S6). Although we attempted to choose viewing fields with similar numbers of cells (8, 12–19) in the four chambers, some variability is unavoidable and the replicative age of the cells may differ in any case. To account for this variability, we normalized the Imd2-GFP induction curves using the timepoint when they reached 50% confluence and found that the induction curves were highly reproducible.

### MPA does not alter the guanine sensitivity of early and late Imd2-GFP induction

Given that Imd2 mRNA synthesis is repressed by high intracellular GTP concentration due to use of nonproductive start sites by RNAP II (Fig. 1), simultaneous addition of MPA and guanine to the growth medium is expected to suppress transcriptional induction. Guanine is converted directly to GMP by Hpt1, bypassing Imd2 and its inhibition by MPA. This expectation has been validated at the mRNA level in the case of yeast cells grown in suspension culture (8, 12, 14). To see if guanine suppression of MPA-dependent Imd2 induction also occurs at the protein level in our microfluidic system, we treated cells with 1.5 µg/mL MPA and increasing concentrations of guanine (0, 100, 200, or 400 µM).

**Fig 4:**
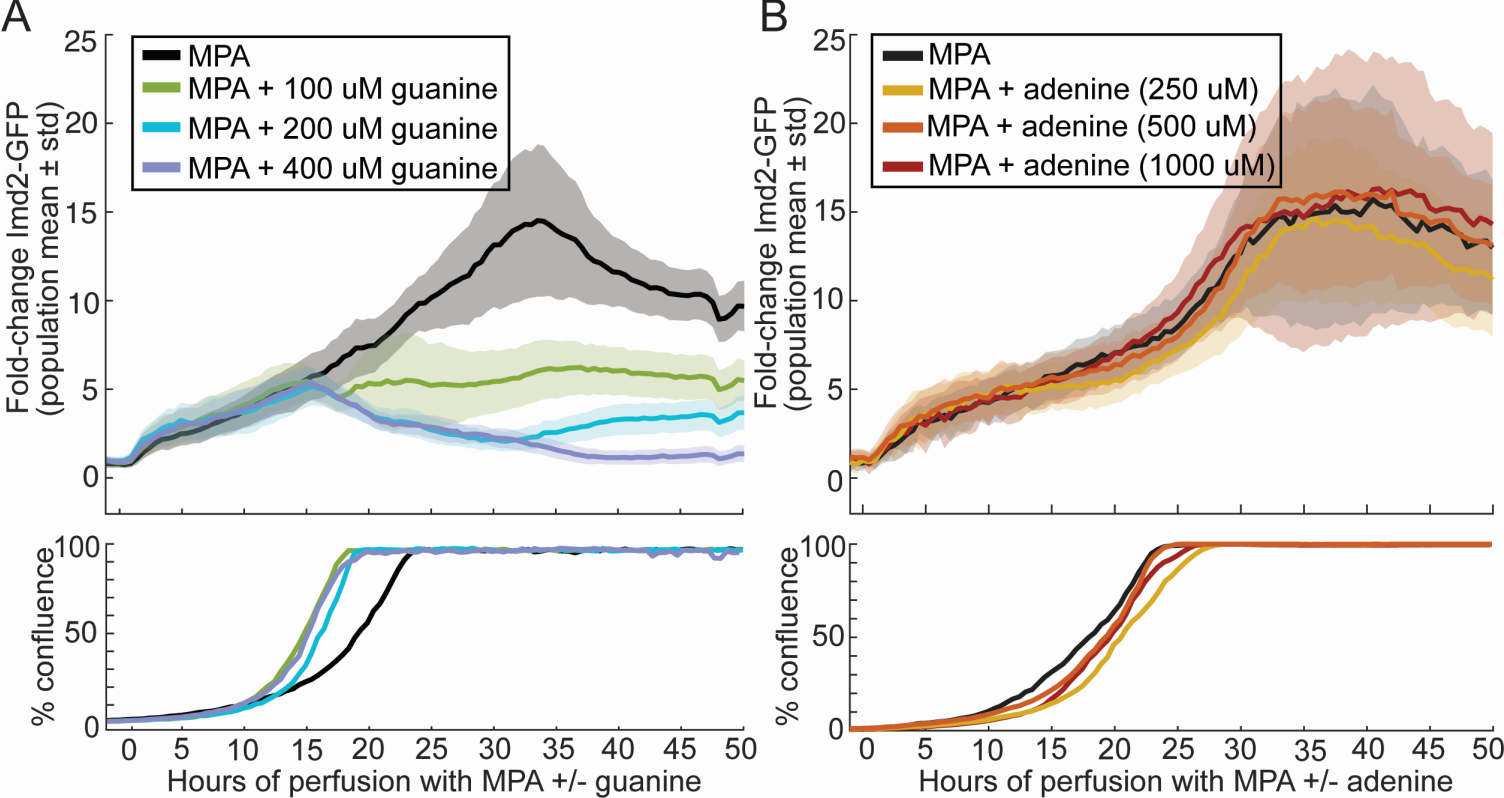
Guanine-specific and dose-dependent suppression of Phase 2 induction of Imd2-GFP in the presence of MPA. (**A**) Top panel: population mean Imd2-GFP fold change (solid lines) with one standard deviation above and below the mean (shaded area) for 1.5 µg/mL MPA alone (black) or in the presence of 100 µM (green), 200 µM (blue), or 400 µM guanine (purple). Bottom panel: percentage of the viewing field occupied by cells over time. (**B**) Same as panel A, but with the indicated concentrations of adenine in place of guanine.

The presence of guanine in the medium had no effect on the approximately fivefold Phase 1 induction of Imd2-GFP during the first 15 hours after the addition of MPA (Fig. 4A). In contrast, Phase 2 of Imd2-GFP induction was suppressed by guanine in a dose-dependent manner, consistent with Phase 2 induction being driven by altered *IMD2* start site selection in response to low GTP. This result shows that the earlier, Phase 1 induction observed in the presence of 1.5 µg/mL MPA is unlikely to be due to low GTP levels. Guanine supplementation decreased the time to reach confluency by about 5 hours, consistent with the expected rescue of guanine nucleotide limitation caused by the MPA (Fig. 4A, bottom panel). Importantly, this increase in growth rate indicates that guanine was able to enter the cells and be converted to GTP during the time associated with Phase 1 induction. Thus, the lack of an effect of guanine supplementation on Phase 1 induction is not due to the inability of cells to utilize guanine early in outgrowth.

Adenine supplementation could potentially increase the intracellular GTP concentration if an increase in adenylate from base salvage increases the flux of IMP through IMPDH, for example, via feedback inhibition of Ade12 (Fig. 1). To test if this is the case, we treated cells with 1.5 µg/mL MPA and increasing concentrations of adenine (0, 250, 500, or 1,000 µM) but did not observe any notable effect on either cell growth or Imd2-GFP induction (Fig. 4B) indicating that adenine supplementation does not increase GTP levels sufficiently to suppress Phase 2 induction in the presence of MPA.

### High Imd2 expression correlates with increased cell growth

**Fig 5:**
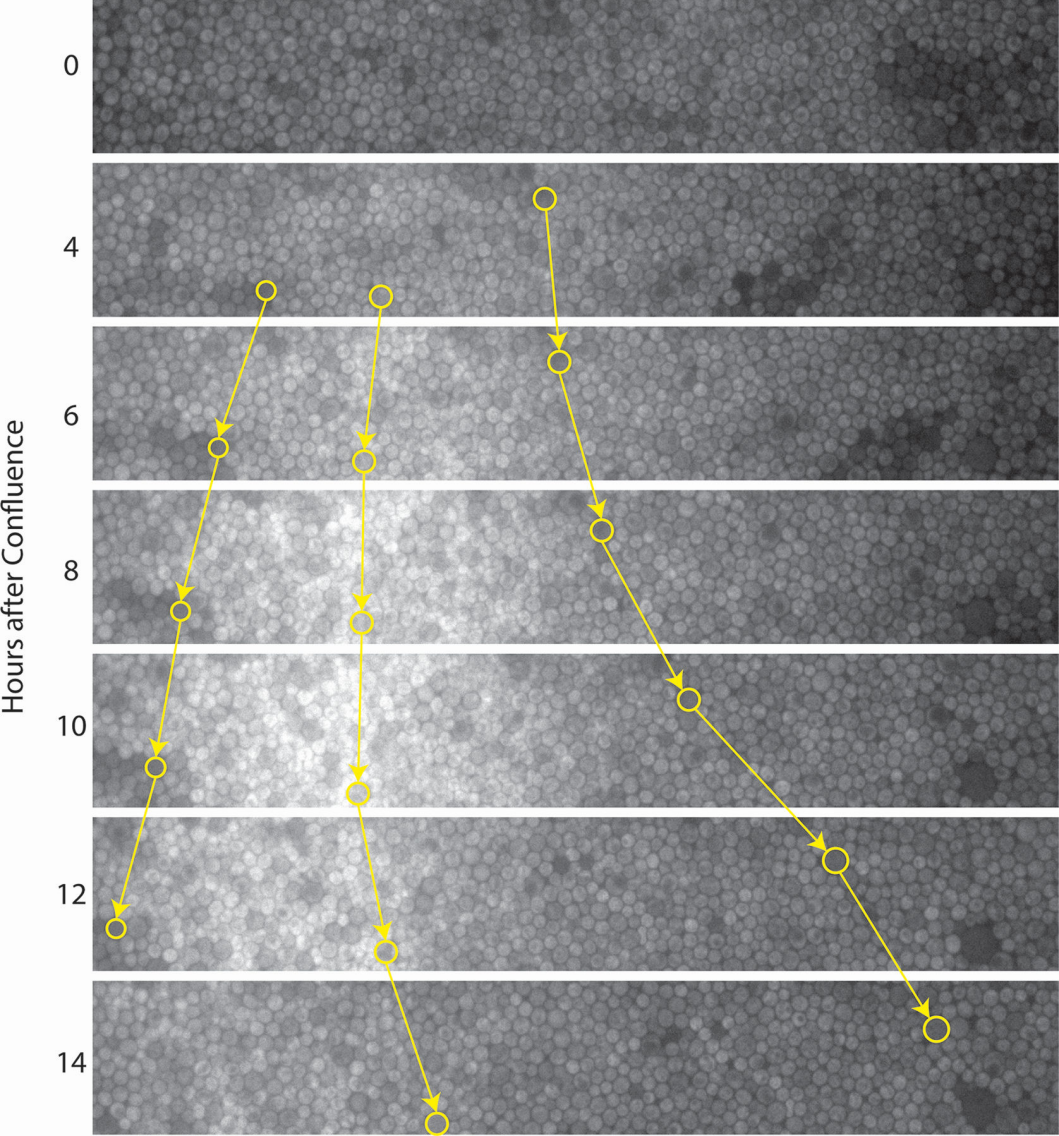
Imd2-GFP expression is not homogenous across the viewing field after confluence, and cell growth emanates from areas with the highest Imd2-GFP levels. Still images showing the central 20% of the viewing field (vertically) for the Imd2-GFP channel of cells treated with 1.5 µg/mL MPA at 0–14 hours after confluence. Nutrient flow is from right to left. Selected cells, identified by their brightness in the RFP channel, are circled in yellow, and their change in horizontal position over time is highlighted by yellow arrows. Corresponds to data shown in Fig. 4A and Video S6.

We observed that Imd2-GFP induction in MPA-treated cells after confluence was not always homogenous across the width of the viewing field (Fig. 5). The peak of Imd2-GFP induction progressed slowly leftward across the viewing field after confluence, away from the nutrient inlet (Fig. 5; Video S6). This Imd2-GFP “wave” collapsed to a roughly homogenous distribution across the viewing field 15 hours after the cells reached confluence.

We further noted that the band of cells with the highest Imd2-GFP signal appeared to be growing most rapidly, based on the observation that RFP-bright “marker” cells were pushed away in both directions from the band of GFP-bright cells (Fig. 5), and that the zone from which cells emanated shifted leftward with the peak of Imd2-GFP (Fig. S7). These observations imply that cell growth is preceded by an increase in Imd2-GFP expression, consistent with increased Imd2 mRNA synthesis prior to entry into the cell division cycle (31). The net movement of cells toward the nutrient source we observe is analogous to growth of a colony outwards from a founder cell on solid medium. Although cells can grow in both directions (left and right) in the microfluidic plate, they presumably grow faster to the right due to the spatial gradient of nutrients.

The wave of Imd2-GFP expression postconfluence was observed for cells treated with MPA and, at a lesser extent, for cells treated with MPA and 100 µM guanine but was not observed for cells treated with MPA and the two highest concentrations of guanine (200 and 400 µM). Thus, the wave is likely due to starvation for guanine in confluent cells that are distal to the nutrient source. We hypothesize that increased expression of Imd2 increases the intracellular GTP concentration and allows the cells to enter S phase and complete the cell cycle. This phenomenon was not clearly observed for every experiment where cells were treated with MPA, possibly due to variability in the distance of the chosen viewing field from the media inlets or in the flow rate of nutrients through the cell mass.

### Overexpression of *IMD2* decreases endogenous Imd2-GFP levels during Phase 2 induction

We investigated if overexpression of *IMD2* from a constitutive promoter on a high copy plasmid decreases accumulation of endogenous Imd2-GFP. This effect could occur by at least two possible mechanisms. Imd2 protein is known to bind polyadenylated RNA transcripts (18) and could potentially regulate its own mRNA in *trans*, for example, by repressing translation. Alternatively, increasing Imd2 protein levels may increase intracellular GTP levels by competing with Ade12 for IMP and thus have an effect similar to supplementation with guanine. To discriminate between these two possibilities, we also tested the effect of overexpression of Imd2 with the Cys335Ala substitution, which renders the enzyme catalytically inactive (32).

**Fig 6:**
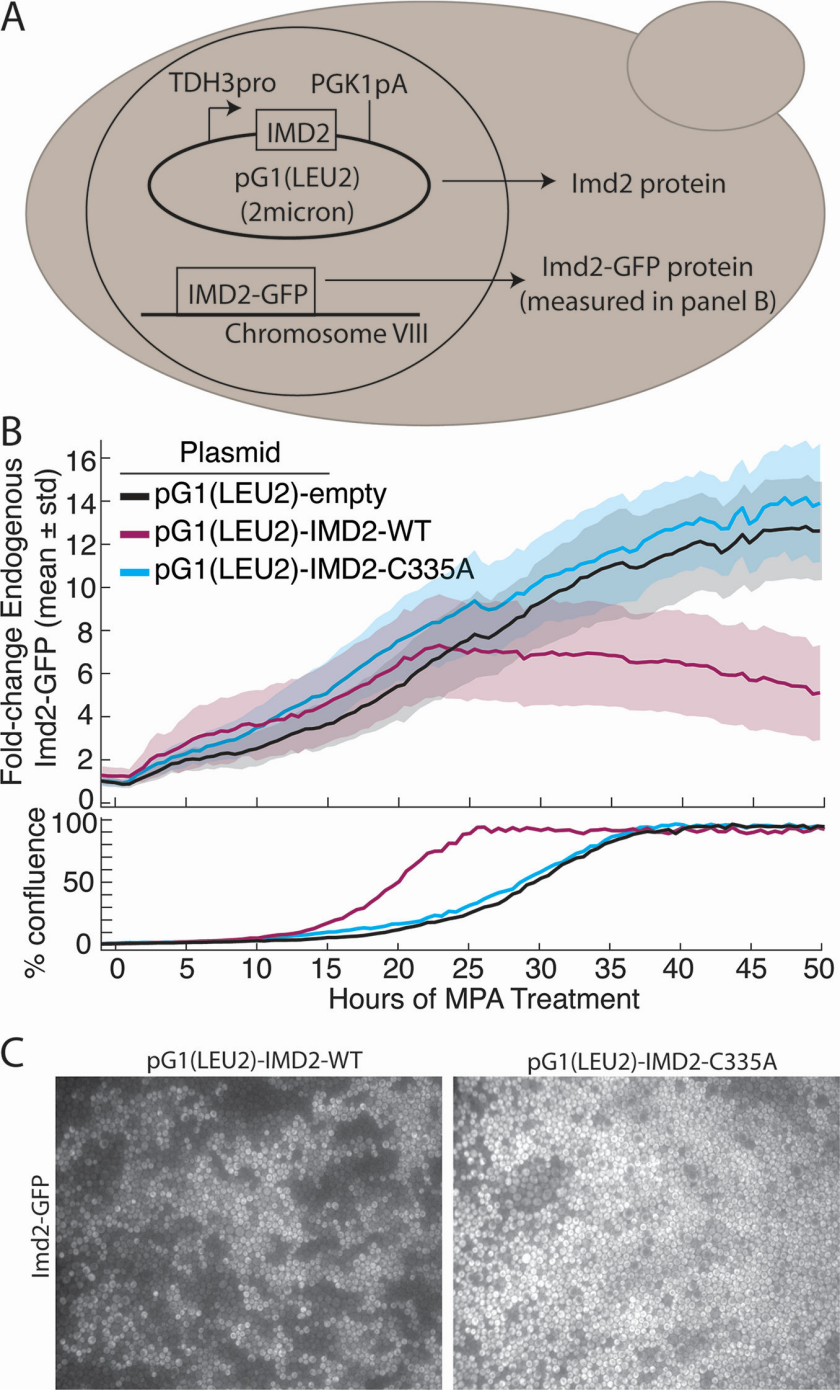
Overexpression of catalytically active *IMD2* from a plasmid decreases endogenous Imd2-GFP levels in confluent cells. (**A**) Schematic genotype of strains used in this experiment. TDH3pro: promoter region from the *TDH3* gene; PGK1pA: 3′-UTR and polyA site from the *PGK1* gene; 2 micron: high-copy yeast plasmid origin. (**B**) Top panel: levels of Imd2-GFP in cells treated with 1.5 µg/mL MPA and containing the pG1(LEU2) plasmid with no insert (black), IMD2-WT (purple), or the catalytically inactive IMD2-C335A allele (blue). Solid lines indicate population mean per cell GFP fluorescence and shading indicates one standard deviation above and below the mean. Bottom panel: the percentage of the viewing field occupied by cells over time. (**C**) Still images of the Imd2-GFP channel when cells expressing the pG1(LEU2)-IMD2-WT or pG1(LEU2)-IMD2-C335A plasmids first reached confluence.

The protein-coding region of *IMD2* with or without the C335A substitution was cloned downstream of the highly expressed and constitutive *TDH3* promoter on a high copy plasmid (Fig. 6A). An “empty” plasmid was also constructed in which the *IMD2* sequence was replaced with a 20-nucleotide linker DNA. Each plasmid was transformed into KES002, and the level of endogenous Imd2-GFP in the presence of 1.5 µg/mL MPA was tracked as before. However, SC-Leu medium was used to maintain selection for the overexpression plasmid, which likely accounts for the longer time to achieve confluence (Fig. 6B).

During Phase 1 induction prior to confluence, the levels of endogenous Imd2-GFP were similar for the empty plasmid and both the wild-type and catalytically dead variants of *IMD2* (Fig. 6B). But after about 23 hours of growth, Imd2-GFP levels in the cells containing the wild-type *IMD2* plasmid started to drop, while in the cells containing the empty vector or catalytically inactive Imd2 plasmid, Imd2-GFP levels continued to increase. These data suggest that the decreased Imd2-GFP levels observed during Phase 2 induction in the presence of the wild-type *IMD2* plasmid are due to increased GTP levels and not a noncatalytic role of Imd2 protein in regulating its own mRNA expression. This conclusion is further supported by the fact that cells containing the wild-type *IMD2* plasmid reached confluence much earlier than cells containing the empty or inactive plasmids, as was the case for cells receiving guanine supplementation (Fig. 4A). We also observed more variable Imd2-GFP expression across the population of cells expressing the wild-type *IMD2* plasmid than for the catalytic mutant strain (Fig. 6C), which is likely due to a variable copy number of the 2-micron plasmid.

### MPA addition to confluent cells results in a rapid induction phase that is abolished by guanine supplementation

While Phase 2 of Imd2-GFP induction correlates well with when cells approach high density, we could not rule out that maximal MPA induction requires some other time-dependent process. If high cell density is the determinant of the second phase of Imd2-GFP induction, then addition of MPA to confluent cells should result in immediate, strong induction that is suppressed by guanine. To test this prediction, we allowed cells to reach high density before exposure to MPA.

**Fig 7:**
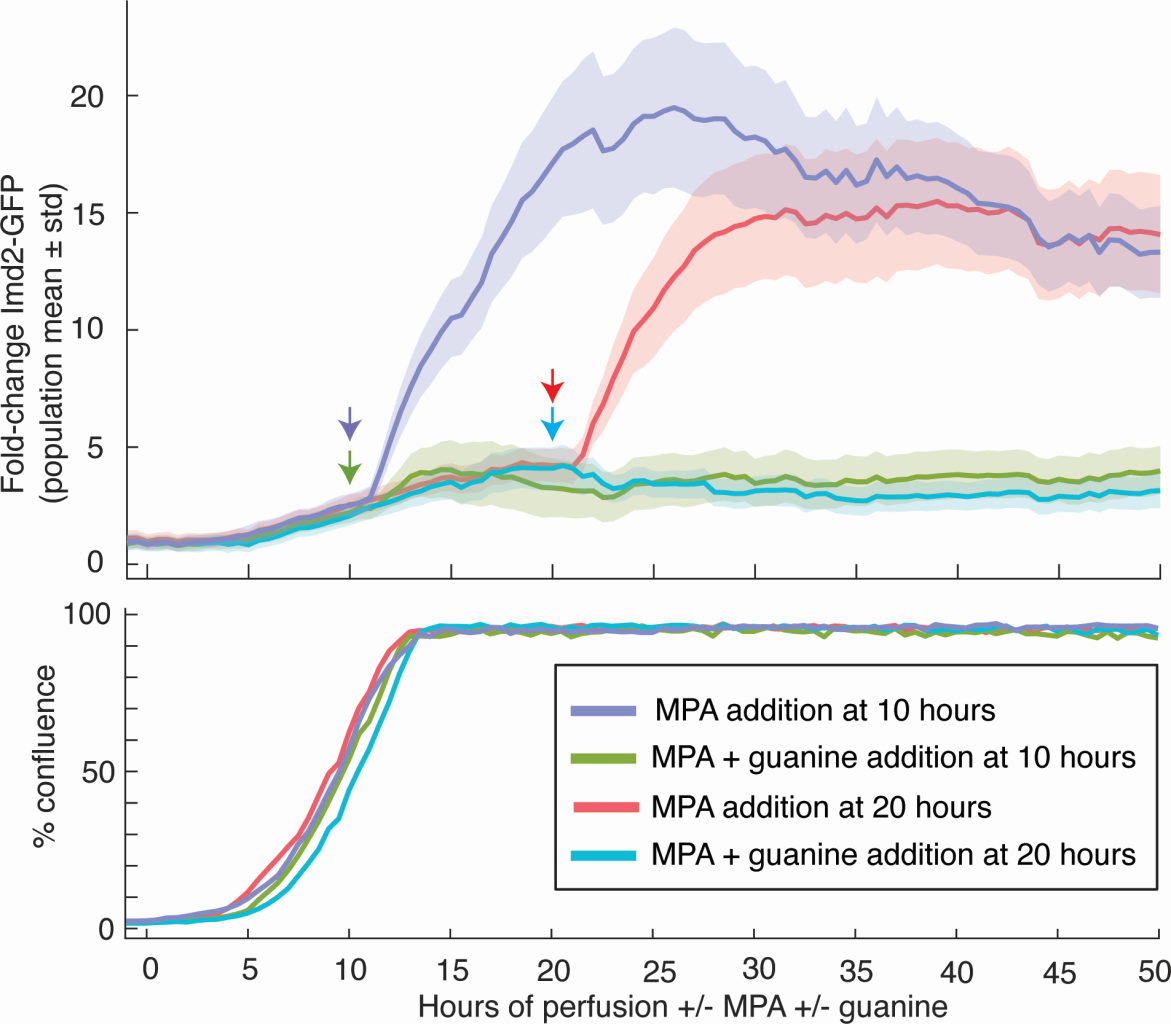
Addition of MPA shortly before or after confluence results in rapid, guanine-sensitive Imd2-GFP induction. Top panel: population mean Imd2-GFP fold induction (solid lines) with one standard deviation above and below the mean (shaded area) for cells perfused for 10 or 20 hours with untreated media before addition of 1.5 µg/mL MPA without or with 400 µM guanine. Colored arrows indicate the time that MPA or MPA + guanine was added to the cells (see key). Bottom panel: the percentage of the viewing field occupied by cells over time.

Indeed, addition of MPA shortly before or well after acquisition of confluence (at 10 or 20 hours, respectively) resulted in a single, strong Imd2-GFP induction phase that was abolished by simultaneous addition of 400 µM guanine (Fig. 7). There was still an approximately 1-hour lag before the induction was observed, similar to cells treated with MPA at 0 hours of perfusion, presumably due to the time required for transcription, translation, and GFP maturation. Furthermore, the amplitude of Imd2-GFP induction (15- to 20-fold) is similar to that seen when MPA is present from 0 hours on. Thus, full Phase 2 induction does not require a prior, MPA-enhanced Phase 1 induction. These results support our conclusion that Imd2 is subject to two induction phases occurring at low or high cell density and regulated by different mechanisms.

### Bypass of the *IMD2* attenuator strongly increases basal Imd2-GFP expression and decreases guanine sensitivity

The only known mechanism by which *IMD2* expression is sensitive to intracellular guanine levels is transcription initiation at the upstream nonproductive “G” start sites under high GTP conditions, resulting in decreased Imd2 mRNA (Fig. 1). We deleted these G start sites (nucleotides −148 through −64 relative to the mRNA transcription start) from the endogenous *IMD2-GFP* locus, which corresponds to a previously described mutation that resulted in increased mRNA from an *IMD2* reporter construct (8).

**Fig 8:**
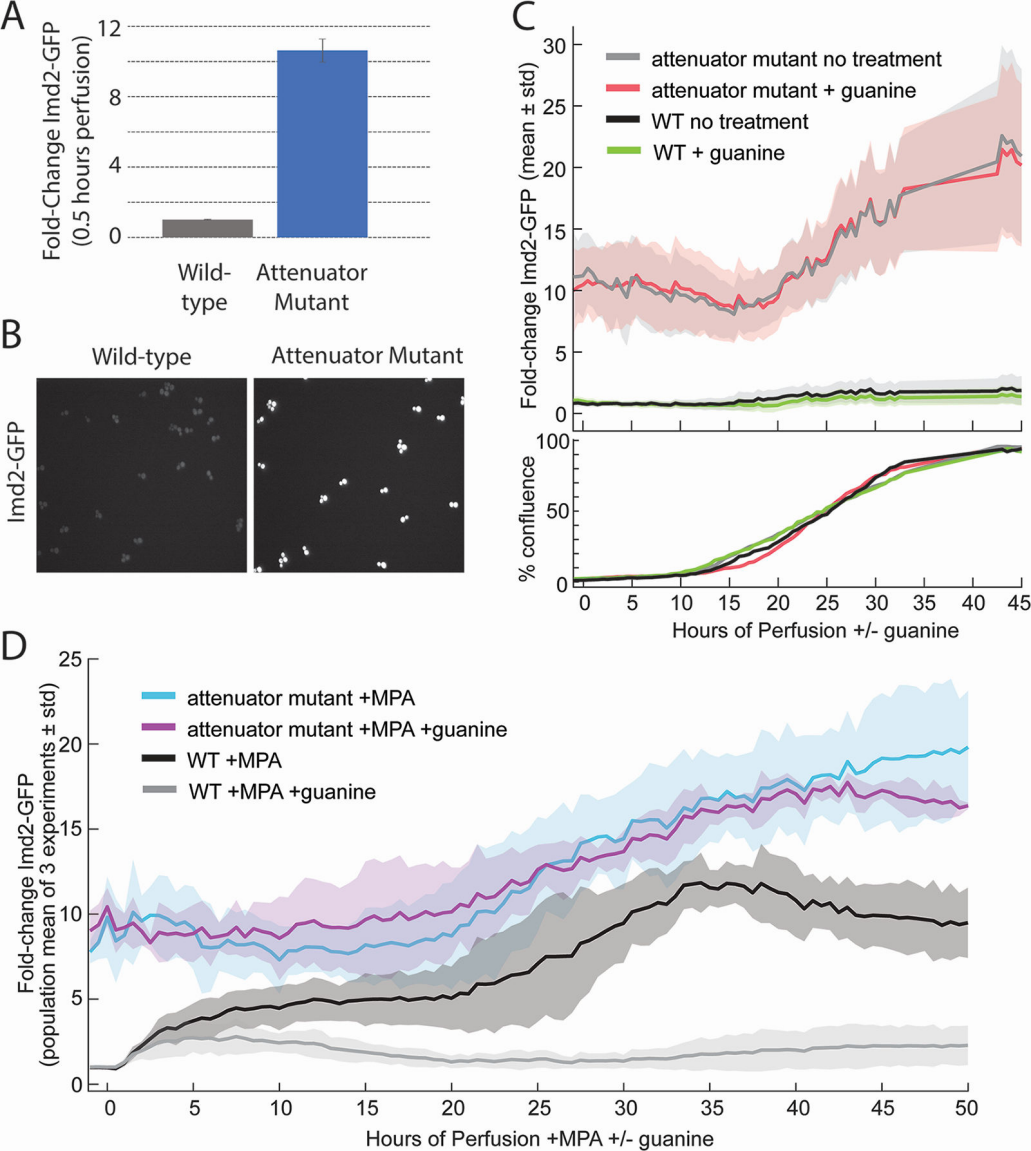
Deletion of nonproductive “G” transcription start sites from *IMD2* increases basal Imd2-GFP levels 10-fold and abolishes guanine sensitivity. (**A**) Imd2-GFP expression in the attenuator mutant strain (ELS107) compared with wild-type cells (KES002) after 0.5 hours of perfusion with untreated media based on seven experiments and at least two technical replicates per experiment. Imd2-GFP levels were normalized to the average Imd2-GFP expression of wild-type cells during the first hour of perfusion with untreated media. Error bars represent standard error of the mean. Based on a two-tailed *t*-test, *P* value = 4 × 10^−12^. (**B**) Images showing the Imd2-GFP channel after 0.5 hours of perfusion with untreated media for wild-type KES002 cells (left) or attenuator mutant ELS107 cells (right). (**C**) Top panel: population mean Imd2-GFP fold induction (solid lines) with one standard deviation above and below the mean (shaded area) for attenuator mutant (ELS107) and wild-type (KES002) cells perfused with untreated media or 400 µM guanine (see key). Bottom panel: the percentage of the viewing field occupied by cells over time. (**D**) Population mean Imd2-GFP fold-induction averaged over three separate experiments (solid lines) with one standard deviation above and below the mean (shaded area) for attenuator mutant (ELS107) and wild-type (KES002) cells perfused with 1.5 µg/mL MPA or MPA with 400 µM guanine (see key).

In the absence of the nonproductive G start sites, Imd2-GFP expression was elevated roughly 10-fold compared with wild-type basal expression levels (Fig. 8A and B). Consistent with GTP sensing occurring via RNAP II start site selection, the attenuator mutant was not sensitive to the addition of guanine and Imd2-GFP levels were nearly identical in untreated and guanine-treated cells (Fig. 8C). Guanine in the presence of MPA also had little effect on Imd2-GFP levels in the attenuator mutant (Fig. 8D). The absence of the G start sites eliminated MPA induction of Imd2-GFP levels (Fig. S8), presumably due to the inability to sense low GTP levels. However, increased accumulation of Imd2-GFP was observed when cells reached about 25% confluence (Fig. 8C, 20 hours). The guanine insensitivity and amplitude of this accumulation suggest that it corresponds to Phase 1 induction occurring later than usual, perhaps due to the high initial level of Imd2-GFP. These results support an exclusive role of the *IMD2* attenuator in conferring GTP-dependent repression of *IMD2* expression and confirm the existence of a guanine-independent mechanism of Imd2 induction.

## DISCUSSION

The accumulation of Imd2 protein in a yeast cell is determined by multiple processes, including mRNA synthesis, mRNA nucleocytoplasmic transport, mRNA translation, mRNA turnover, and protein turnover. In this study, we tracked the endpoint of *IMD2* gene expression, Imd2 protein accumulation, in live cells over 2 days of growth in a constant-flow microfluidic device. We were thus able to confirm and characterize the known mechanism of Imd2 feedback regulation by GTP and, for the first time, observe spatial patterns of Imd2 accumulation in a growing yeast colony. We also uncovered an uncharacterized GTP-independent mode of induction. This approach should be applicable to any protein that tolerates a fluorescent tag well and whose regulation is influenced by diffusible substances and/or cell growth states. Below, we consider the implications of our results for understanding the physiological regulation of IMPDH and GTP levels in cells.

### Guanine-sensitive induction of Imd2 synthesis

Some cellular uses of GTP alter only its phosphorylation state and so do not consume guanylate. For example, GTP is converted to GDP twice for every amino acid inserted into a growing polypeptide, by EF-1α and EF-2, but the GDP is converted back to GTP by nucleotide kinase. Thus, the translation cycle requires a sufficient level of guanylate but does not alter that level. In contrast, RNA and DNA syntheses consume guanylate by incorporating it into nucleic acid. Thus, demand for guanylate synthesis is likely highest in late G1 and early S phases of the cell cycle when cells are making ribosomal RNA and tRNA for cell growth and converting guanylate into deoxyguanylate for DNA synthesis. We observed a “wave” of increased Imd2-GFP accumulation in a confluent yeast cell mass that moved in the direction of the nutrient flow (Fig. 5). The peak of the wave exhibited active cell division, since flanking cells were pushed outwards in both directions (against and with the nutrient flow). The wave of Imd2-GFP was not observed in the presence of sufficient supplemental guanine, indicating that it was induced by guanylate deficiency. Cells that grew from the peak Imd2-GFP region toward the nutrient flow became dimmer in the GFP channel, presumably due to increased access to nutrients, while the cells that grew away from the nutrient flow remained bright, resulting in movement of the GFP-bright zone away from the nutrient inlets. Ultimately, the Imd2-GFP wave flattened as cells far from the nutrient inlet became quiescent or senescent. These results are consistent with Imd2 becoming limiting for guanylate synthesis in cells that are receiving insufficient nutrients. This block to cell growth can be overcome either by increasing Imd2 synthesis or by conversion of supplemental guanine into guanylate by Hpt1 (Fig. 1).

The conditions created artificially in the microfluidic apparatus by the unidirectional nutrient flow are analogous to what a yeast colony might encounter in its natural environment. Yeast cells growing on a solid substrate can be morphologically distinct depending on the proximity of the cell to nutrients on the substrate (20). Furthermore, these phenotypic differences have been associated with the differential expression of a variety of metabolic enzymes, which can result in specialized metabolic profiles of individual cells (20, 21). For example, cells grown in glucose-limited conditions can develop into two classes of cells: one undergoing gluconeogenesis to produce glucose and the other importing glucose for glycolysis (19, 21). This sharing of metabolites within a yeast colony has been documented in other contexts and is beneficial for whole colony survival (22).

Although past studies focused on glucose metabolism during colony development, colonies must also regulate nucleotide metabolism in response to nutrient limitation. Our observations of Imd2 expression in yeast grown in a microfluidic system show that accumulation of a nucleotide biosynthetic enzyme, IMPDH, correlates both with cell density and distance from the nutrient source. Also, induction of Imd2-GFP accumulation by IMPDH inhibition with MPA is much more rapid and robust when cells are near or at confluence than when they are first seeded (Fig. 7), consistent with confluent cells existing in a guanylate-limited state. The 10-fold increase in basal Imd2-GFP accumulation upon deletion of the *IMD2* attenuator and the insensitivity of expression of this allele to guanine supplementation (Fig. 8) confirms that the attenuator is the primary sensor of the intracellular guanylate level with respect to Imd2 protein synthesis.

### Guanine-insensitive induction of Imd2 synthesis

The guanine-sensitive induction of Imd2 discussed above is seen when cells are nearing confluence, consistent with decreased access to nutrients due to consumption by upstream cells and/or physical blockage of fluid flow by the cell mass. What then might be the signal for guanine-insensitive induction, which occurs when cells are still sparse in the viewing field? Our observation that Phase I induction occurs earlier in the presence of MPA but is still guanine insensitive suggests that some effect of MPA other than decreased GTP level is operative. Given that MPA traps a covalent enzyme-substrate intermediate, E-XMP* (33, 34), one possibility is that this arrested form of Imd2 does not turnover as rapidly and thus accumulates to higher levels. Still, since guanine-independent induction is also seen in the absence of MPA, it cannot simply be an artifact of MPA treatment. It is possible that cells in the viewing field are responding to signaling molecules released by cells upstream in the flow. Cells that accumulate at the constriction between the monolayer and the media inlet during cell loading achieve confluence early and may stimulate outgrowth of downstream cells with a secreted molecule that flows into the viewing field. Such a cell-cell signaling pathway would be distinct from induction due to guanine starvation, which is cell autonomous. Another possibility is that constraint of the yeast cells by the ceiling and floor of the monolayer region somehow induces higher Imd2 expression than what occurs in cells in suspension, perhaps due either to physical forces sensed by the cytoskeleton or to decreased uptake of small molecules (other than guanine) into the cell at the substrate interface. This latter signaling mechanism could be an adaptation to growth in a multilayer colony on a surface.

What might be the outcome of the proposed guanine-insensitive signaling pathways? An obvious possibility is increased RNAP II recruitment to the *IMD2* promoter, which would increase the number of scanning preinitiation complexes and transcription initiation at all start sites, including the mRNA start site. However, posttranscriptional regulation is also possible. Further studies will be required to determine the mechanism of this newly discovered, guanine-insensitive induction of Imd2.

### Potential insights from real-time observation of yeast cell behavior in a growing colony

The ability to view living yeast cells as they form a monolayer colony and observe their budding, cell morphology, and molecular markers such as Imd2-GFP and Nhp6A-RFP provides unique insight into their behavior. Here, we further note two incidental observations we made viewing yeast cells for 2 days in the microfluidic apparatus, which suggests this approach could potentially provide insight into cell senescence and cell-cell communication.

The first is a rare behavior observed in the no-MPA channel of the experiment shown in Fig. 3 (see Video S7). A large cell near the middle of the right border produces several elongated buds that soon die (as judged by cytosolic Nhp6A-RFP signal), after which the mother cell develops a long protrusion and stops budding. This may be a mother cell experiencing necrotic death (24).

The second is the observation of apparent connections between yeast cells in a confluent mass, visible in high magnification images (for example, Fig. S4A) and occasionally in time-lapse videos (for example, Video S7, as the mass of cells enters from the right). While we cannot exclude that these apparent connections are an optical artifact, it is possible that constraint to a monolayer exposes existing cell-cell connections. These inter-cell connections may be the same as those seen after selection of “snowflake yeast,” which are thought to be due to incomplete mother-daughter cell separation (35), as seen previously after MPA treatment (30).

We suggest that time-lapse microscopy studies of yeast colony development in selected mutant strains could provide insight into the mechanisms of adaptations to colony growth. Other investigators are pursuing this approach using custom microfluidic cells with geometries that allow individual cell lineages to be followed over time (see, for example, 36).

## MATERIALS AND METHODS

### Oligonucleotides

See Table S1. Oligonucleotides were ordered from Integrated DNA Technologies.

### Plasmids

#### pRS416-Cas9-86fw

Plasmid pR416-TEF1p-Cas9-NLS-crRNA-BaeI (37) was digested with BaeI and ligated to kinase-treated and annealed oligos 1642 and 1643.

#### pRS315-IMD2(−702/+512)

*IMD2* sequence from bp −702 to +512 relative to the mRNA transcription start site of *IMD2* was PCR amplified using KES002 genomic DNA and oligos 1644 and 1645, which also added a SacI restriction site to the 5′ end of the fragment and a SpeI restriction site to the 3′ end of the fragment. The PCR fragment was digested with SacI and SpeI and ligated into a SacI/SpeI digested pRS315 vector.

#### pRS315-IMD2Δ(−148/−64)-repair

Base pairs −148 through −64 of *IMD2*, relative to the mRNA transcription start site, were deleted from the pRS315-IMD2(−702/+512) plasmid by using inverse PCR with oligos 1646 and 1647. An EcoRI site was also added by mutating bp (−160) to (−162), relative to the transcription start site, from AAT to CCT.

#### pG1(LEU2)-IMD2

To create a plasmid for the constitutive expression of Imd2, the *IMD2* ORF was inserted downstream of the *TDH3* promoter and upstream of the *PGK1* 3′-UTR and pA site in a high-copy yeast shuttle vector. *IMD2-GFP* was amplified from KES002 genomic DNA by PCR using oligos 1476 and 1477, followed by PCR amplification of the *IMD2* coding region alone using oligos 1558 and 1559, which contain SacI and SalI restriction sites. This two-step strategy was used to eliminate the possibility of amplifying the other *IMD* genes. The resulting PCR product was digested with SacI and SalI and cloned into the same sites of a variant of the pG1 2-micron vector (38) that had the *TRP1* marker replaced with *LEU2*, named pGAC24 (39). This cloning replaced the *ACT1-CUP1* reporter gene in pGAC24 with *IMD2*. QuikChange mutagenesis using oligos 1568/1569 was used to restore the endogenous *IMD2* stop codon.

#### pG1(LEU2)-empty

To create a control for the experiments in which *IMD2* was constitutively expressed from pG1(LEU2) plasmids in yeast, the *ACT1-CUP1* reporter was replaced with a 20-base pair sequence determined by a random DNA sequence generator (http://www.faculty.ucr.edu/~mmaduro/random.htm). Oligos 1536/1537 were annealed and kinased before ligation into SacI/SalI-digested pGAC24.

#### pG1(LEU2)-IMD2-C335A

To create a plasmid for the constitutive expression of a catalytically dead variant of Imd2 containing the C335A mutation in yeast (equivalent to C331A in human IMPDH1; 32), pG1(LEU2)-IMD2 was mutagenized by QuikChange mutagenesis using oligos 1306/1307 to mutate nucleotides T1003>G and G1004>C.

### Yeast strains

The Imd2-GFP strain we designate KES000 [*MAT**a** his3Δ1 leu2Δ0 met15Δ0 ura3Δ0 IMD2::GFP(S65T)/HIS3*] was purchased from Invitrogen (#95700) and derives from strain BY4741 (40). Plasmids were transformed into yeast by the lithium acetate procedure (41).

### KES002

(*MAT**a** his3Δ1 leu2Δ0 met15Δ0 ura3Δ0 IMD2::GFP(S65T)/HIS3; NHP6A::TagRFP-T/nat*)

The HindIII/XbaI fragment of pRS315-NHP6A-TagRFP-T/nat (42) that contains a *NHP6A*-TagRFP-T fusion gene derived from EY2426 (23) was gel purified and transformed into KES000. Integrants were selected by plating onto YEPD containing 100 µg/mL nourseothricin (also called cloneNAT or streptothricin; from Axxora or US Biologicals). Chromosomal integration of the NHP6A-TagRFP-T/nat DNA was confirmed by PCR with flanking oligonucleotides and sequencing.

### ELS107

(*MAT**a** his3Δ1 leu2Δ0 met15Δ0 ura3Δ0 IMD2(Δ*-148/–64)*:: GFP(S65T)/HIS3; NHP6A::TagRFP-T/nat*)

500 ng of pRS416-Cas9-86fw and 5 µg of pRS315-IMD2Δ(–148/–64)-repair were transformed into KES002 that was treated or not with 1.5 µg/mL MPA overnight and for an additional 3 hours before making the cells competent. Treatment with MPA was to test if increased transcription of *IMD2* would enhance Cas9-sgRNA targeting to the locus. After incubation with DNA, cells were plated on selective medium (Sunrise Science CSM-Leu-Ura and Difco yeast nitrogen base without amino acids) that also contained 1.5 µg/mL MPA. Transformants were then grown in YEPD medium overnight and plated on SC medium containing 1 mg/mL 5-FOA to select against the pRS416-Cas9-86fw plasmid. Twelve colonies each from the MPA-treated or untreated transformants were then screened for the deletion of base pairs −148 to −64 by PCR amplification of genomic DNA using oligos 1589 and 1476 and digestion of the PCR product using EcoRI to detect a restriction site unique to the repair DNA. PCR products that were digested with EcoRI were sequenced verified. Four of 12 transformants from the MPA-treated cells contained the deletion whereas 0 of 12 transformants from untreated cells did, suggesting that increased transcription may increase the frequency of Cas9-initiated gene conversion at the *IMD2* locus.

### Yeast cell culture for microscopy

A single colony of yeast from a YEPD plate was grown at room temperature overnight in sterile, 0.2-μm filtered SC medium. SC medium contains 6.6% (wt/vol) yeast nitrogen base without amino acids (Difco) with 1× SC (Hopkins) supplement mixture (Sunrise Scientific) and 2% glucose. Before loading onto the microfluidic plate, the overnight yeast culture was diluted to 0.2 OD_600_ in fresh SC medium and grown at room temperature for approximately 3 hours until the cells reached mid-log phase (0.4–0.8 OD_600_). If necessary, cells were diluted to 0.4 OD_600_ before loading onto the microfluidic plate.

Mycophenolic acid (from Sigma or Alexis/Fisher), adenine (Sigma), and guanine (99+%, Acros Organics) solutions were made by first dissolving the compounds in 0.1 M NaOH to a concentration of 5 mg/mL for MPA, 50 mM for adenine, or 25 mM for guanine. The stock solution was then diluted in SC media to the appropriate concentration. A volume of 0.1 M HCl equal to the added stock solution was added to neutralize the pH of the resulting solution before perfusing cells. DAPI (Sigma) was added to SC media to a final concentration of 2.5 µg/mL. Calcofluor white (Sigma, 1 g/L) was diluted 1:100 in PBS (137 mM NaCl, 2.7 mM KCl, 10 mM Na_2_HPO_4_, and 1.8 mM KH_2_PO_4_).

### Microscopy of live yeast in a microfluidic platform

Live cell microscopy was done with a CellASIC ONIX system using Y04C-02 plates and attached to a Nikon Eclipse Ti inverted microscope controlled with NIS-Elements software. Images were captured with an Andor Clara camera. The CellASIC Onix 2 system was used for all experiments except the ones shown in Fig. 4B and Fig. S6, which were acquired using the CellASIC Onix 1 system. Temperature was maintained at 30°C using an Okolab microscope cage incubator. Before loading cells, all media inlets that would be used during the course of the experiment were washed with SC medium (see above) perfused at 5 psi for 5 minutes. Cells were then loaded at 4 psi for 5 seconds followed by washing with SC media at 5 psi for 5 minutes to remove any loose cells. The viewing field of a 40× objective was chosen by finding 12–20 cells close to the midpoint in the y dimension of the chamber with the lowest ceiling height and close to the nutrient inlet. After loading the cells, SC medium was perfused for 1 hour before switching to SC medium with additives (if any) at time zero. All perfusions were performed at 0.5 psi except for the following experiments perfused at 1 psi (Fig. 4B, 8C and D, and Fig. S6).

Unless indicated otherwise, images were taken through a 40× objective (Nikon MRD00400; N.A. 0.95) every 30 minutes in each of the four viewing fields by DIC and epifluorescence using GFP (470/40×, 525/50 m) and mCherry (560/40×, 630/75 m) filters (Chroma) with exposure times of 20 ms for DIC, 500 ms for GFP, and 1 s for mCherry. For DAPI and calcofluor-white epifluorescence, a DAPI filter (350/50×, 460/50 m) (Chroma) was used with 500 ms exposure time. The autofocus and z coordinates for each viewing field were manually adjusted at approximately 5–8 hours and 15–20 hours after the start of the experiment to ensure that appropriate focus was maintained over the course of the experiment. A single focal plane was used for imaging, corresponding approximately to the midplane of the cells.

### Quantification of Imd2-GFP expression in live cells

Cells were identified and their GFP fluorescence measured from multichannel timelapse microscopy images using a custom MATLAB pipeline (https://github.com/mccleanlab/cell_finder, Fig. S1). Bio-Formats was used to load ND2 images directly into MATLAB (43), and cells were identified from either the DIC or GFP channels based on a manual evaluation of image quality. In either case, the images were processed with a Laplacian of Gaussian filter to enhance cell contrast and circular regions of interest (ROIs) likely containing individual yeast cells were identified using MATLAB’s circle finder. The mean GFP fluorescence of each ROI was measured from the original (nonenhanced) images. ROIs with fluorescence values below a manually selected threshold were excluded as false positives. Percent confluency was calculated from the area occupied by the retained ROIs. The number of identified cells in the first timepoint ranged from 12 to 71 and in the last timepoint ranged from 2,405 to 2,900.

MATLAB and the GRAMM data visualization toolbox (44) were used to plot the cell measurements. The background GFP signal, quantified as the mode pixel value of the first 10 timepoints, was subtracted from all GFP measurements before plotting, and unless otherwise noted, fold change GFP was calculated based on the mean cellular GFP level across all conditions before treatment. All graphs represent a single experiment with a four-channel CellASIC plate except for Fig. 8D, which represents the average of three experiments.

## Data Availability

Timelapse image stacks and associated metadata for all experiments are available at Dryad (https://datadryad.org) with the DOI: 10.5061/dryad.80gb5mkzb.

## SUPPLEMENTAL MATERIAL

Supplemental material mbio.01021-24-s0001.pdf Fig. S1-S8, Table S1, and video legends.

Video S1 mbio.01021-24-s0002.mp4 KES002 yeast cells grown in SC media without additional treatment (biological replicate B from Fig. 2C).

Video S2 mbio.01021-24-s0003.mp4 KES002 yeast cells grown in SC medium and treated with 400 μM guanine (biological replicate B from Fig. 2C).

Video S3 mbio.01021-24-s0004.mp4 KES002 yeast cells grown in SC medium and treated with 1.5 μg/mL MPA.

Video S4 mbio.01021-24-s0005.mp4 KES002 yeast cells grown in SC medium without additional treatment for 50 hours and then perfused with 2.5 μg/mL DAPI in medium for 160 min.

Video S5 mbio.01021-24-s0006.mp4 KES002 yeast cells grown in SC medium without additional treatment (biological replicate A).

Video S6 mbio.01021-24-s0007.mp4 KES002 yeast cells grown in SC medium and treated with 1.5 μg/mL MPA.

Video S7 mbio.01021-24-s0008.mp4 KES002 yeast cells grown in SC medium without additional treatment.

ASM does not own the copyrights to Supplemental Material that may be linked to, or accessed through, an article. The authors have granted ASM a non-exclusive, world-wide license to publish the Supplemental Material files. Please contact the corresponding author directly for reuse.
