# Supplementary material for: Live-cell analysis of IMPDH protein levels during yeast colony growth provides insights into the regulation of GTP synthesis
Source: mBio. 2024 Jun 28;15(8):e01021-24. doi: 10.1128/mbio.01021-24 (PMC11323793; doi:10.1128/mbio.01021-24)
Supplement: Supplemental material — Fig. S1-S8, Table S1, and video legends. [file mbio.01021-24-s0001.pdf]

SUPPLEMENTAL MATERIALS

The supplemental materials consist of eight figures, one table, and seven videos. All videos are at 3 frames per second with each frame corresponding to 30 min elapsed time.

Video 1. Time lapse Video of KES002 yeast cells grown in SC media without additional treatment (biological replicate B from Figure 2C). Images were taken every 30 minutes. The DIC channel is shown in gray, the GFP channel is shown in green, and the RFP channel is shown in red. Note that the RFP signal is generally dimmer than the GFP signal. Cells that appear yellow are bright in both the RFP and GFP channels and are typically misshapen.

Video 2. Time lapse Video of KES002 yeast cells grown in SC media and treated with 400 μM guanine (biological replicate B from Figure 2C) after 1 hour of being loaded into the CellASIC chamber. DIC channel is shown in gray, the GFP channel is shown in green, the RFP channel shown in red.

Video 3. Timelapse Video of KES002 yeast cells grown in SC media and treated with 1.5 μg/mL MPA 1 hour after being loaded into the CellASIC chamber and the first image taken. Corresponds to data shown in Figure 3. DIC channel is shown in gray, Imd2-GFP channel is shown in green, and Nhp6a-RFP is shown in red. Note the strong induction of Imd2-GFP.

Video 4. Timelapse Video of KES002 yeast cells grown in SC medium without additional treatment for 50 hours and then perfused with 2.5 μg/mL DAPI in media for 160 min. Only the DAPI infusion period is shown. A frame was taken each 5 min starting at DAPI addition. The frame rate is 2/sec. Medium flow is from right to left.

Video 5. Timelapse Video of KES002 yeast cells grown in SC media without additional treatment (biological replicate A). Corresponds to data shown in Figure 2. DIC channel is shown in gray, the GFP channel is shown in green, and Nhp6a-RFP is shown in red. Images were taken every 30 minutes. A “blowout” of cells occurs between 20 and 20.5 hours of perfusion.

Video 6. Timelapse Video of KES002 yeast cells grown in SC media and treated with 1.5 μg/mL MPA after 1 hour of being loaded into the CellASIC chamber and the first image taken. Corresponds to data shown in Figure 4A (without guanine). DIC channel is shown in gray, Imd2-GFP channel is shown in green, and Nhp6a-RFP is shown in red. A peak of Imd2-GFP brightness and cell growth moves leftward after cells reach confluence.

Video 7. Timelapse Video of KES002 yeast cells grown in SC media without additional treatment. Corresponds to data shown in Figure 3. DIC channel is shown in gray, Imd2-GFP channel is shown in green, and Nhp6a-RFP is shown in red.

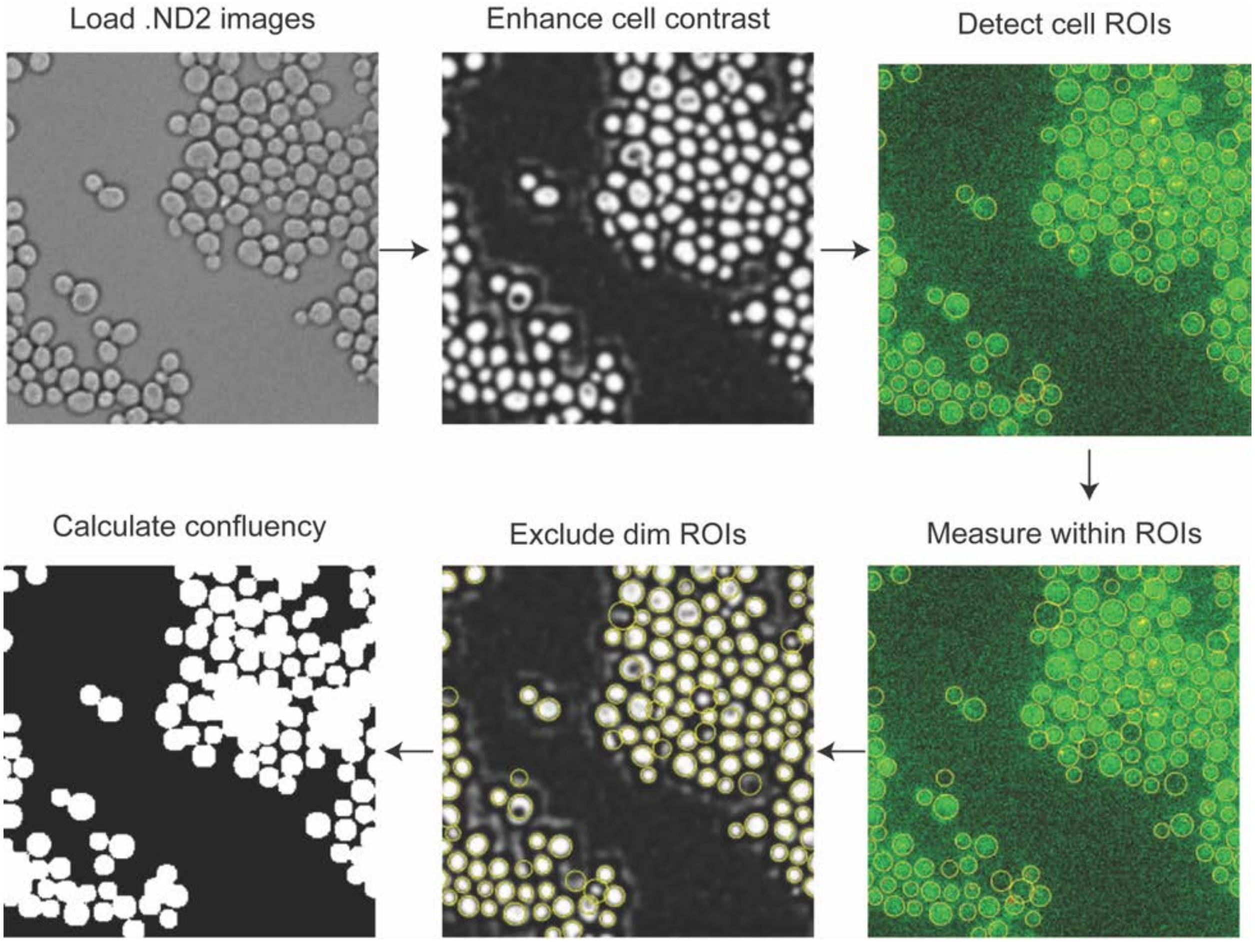

Supplemental Figure 1. Workflow for the identification of cells from microscopy images and calculation of GFP levels. .ND2 images were loaded directly into MATLAB and cells were identified from either the DIC or GFP channels based on a manual evaluation of image quality. Images were processed with a Laplacian of Gaussian filter to enhance cell contrast and circular regions of interest (ROIs), shown as yellow circles, likely containing yeast cells were identified using MATLAB's circle finder. The mean GFP fluorescence of each ROI was measured from the original (non-enhanced) images. ROIs with fluorescence values below a manually selected threshold were excluded as false positives. Confluency was calculated from the area occupied by the retained ROIs.

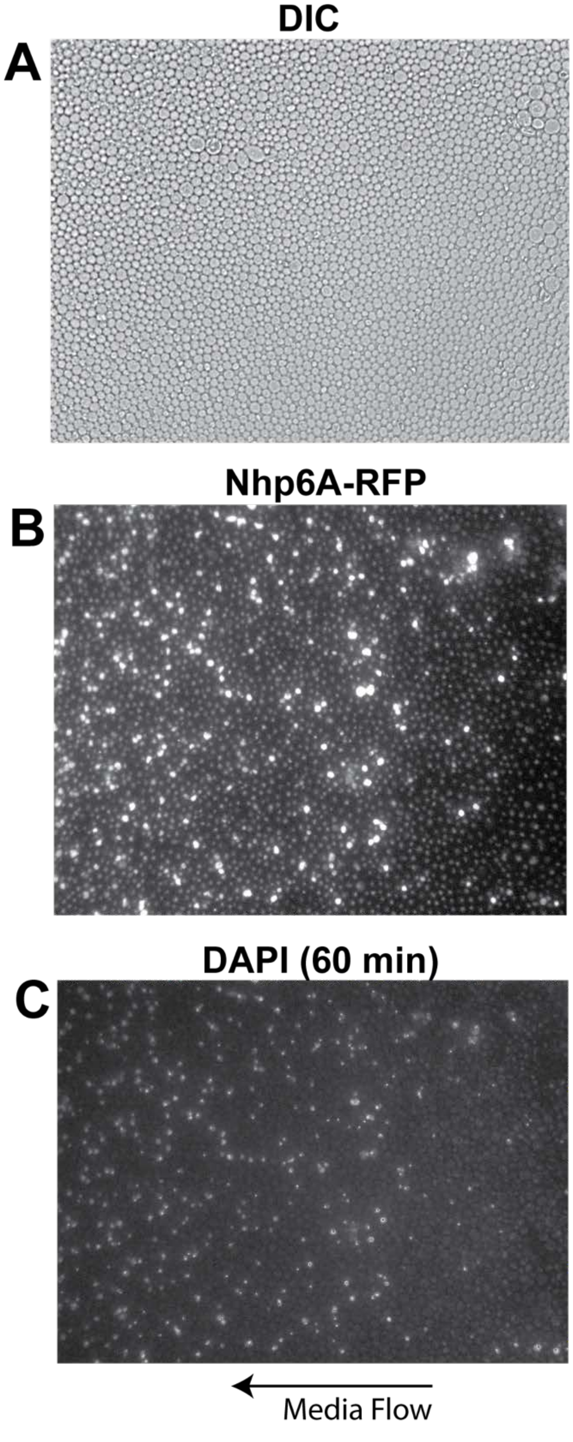

Supplemental Figure 2. Perfusion of 2.5 μg/mL DAPI in media after 50 hours of perfusion with SC media. (A) DIC image of cells after 50 hours of perfusion just before DAPI perfusion. (B) Nhp6a-TagRFP-T (chromatin marker) fluorescence just before DAPI perfusion. (C) DAPI image after 60 minutes of DAPI perfusion (see Supplemental Movie 4 for time course).

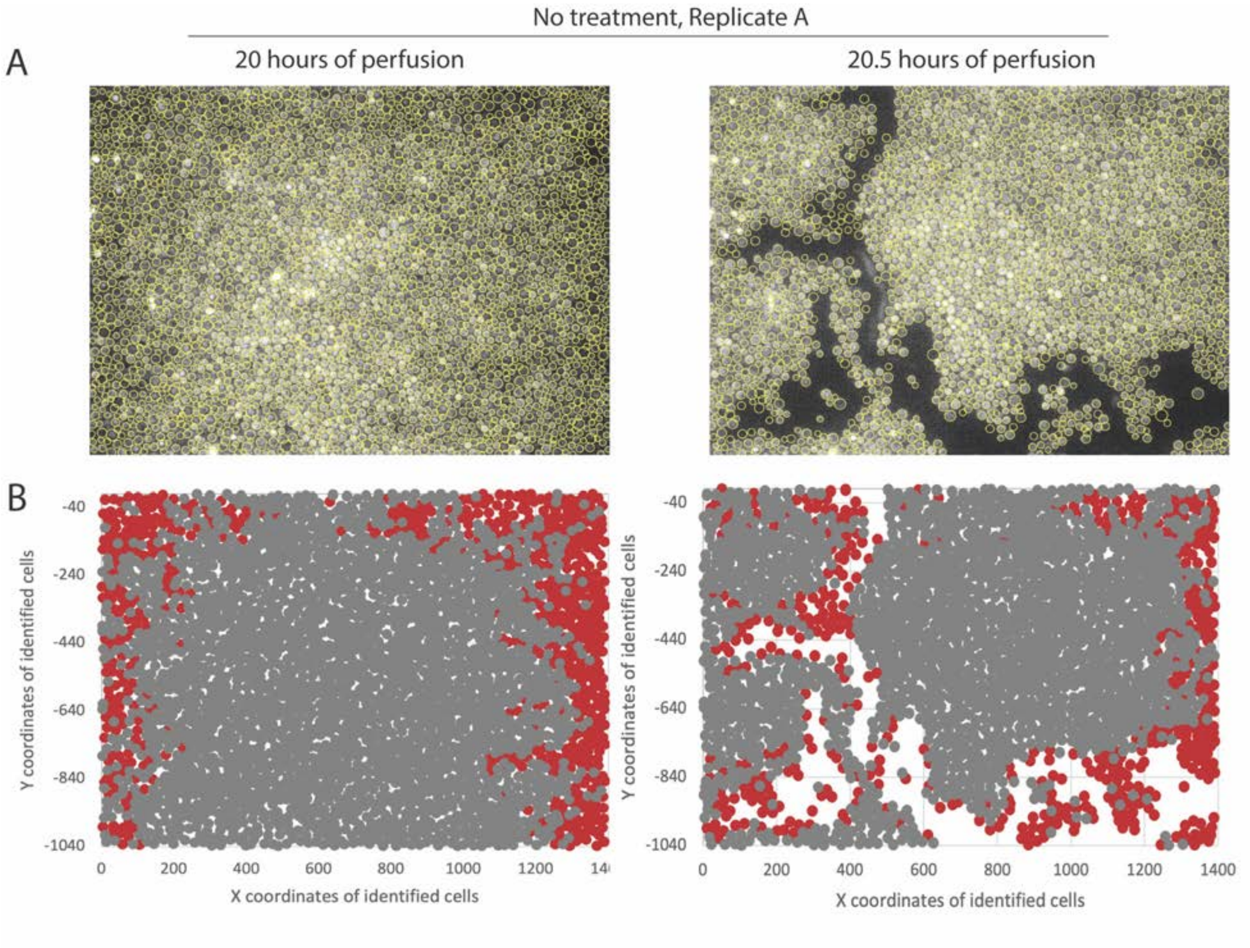

Supplemental Figure 3. Medium incursion into confluent cells results in reduced Imd2-GFP induction for cells in contact with the medium. (A) Still images of Imd2-GFP expression in Replicate A from Figure 2C after 20 hours (*left panel*) or 20.5 hours (*right panel*) of perfusion with SC media without additions. Yellow circles indicate identified regions of interest (ROIs) that are considered as individual cells. (B) X and Y coordinates of identified cells from the images in Panel A are indicated as gray circles, with those in the lowest 20% of Imd2-GFP expression highlighted in red.

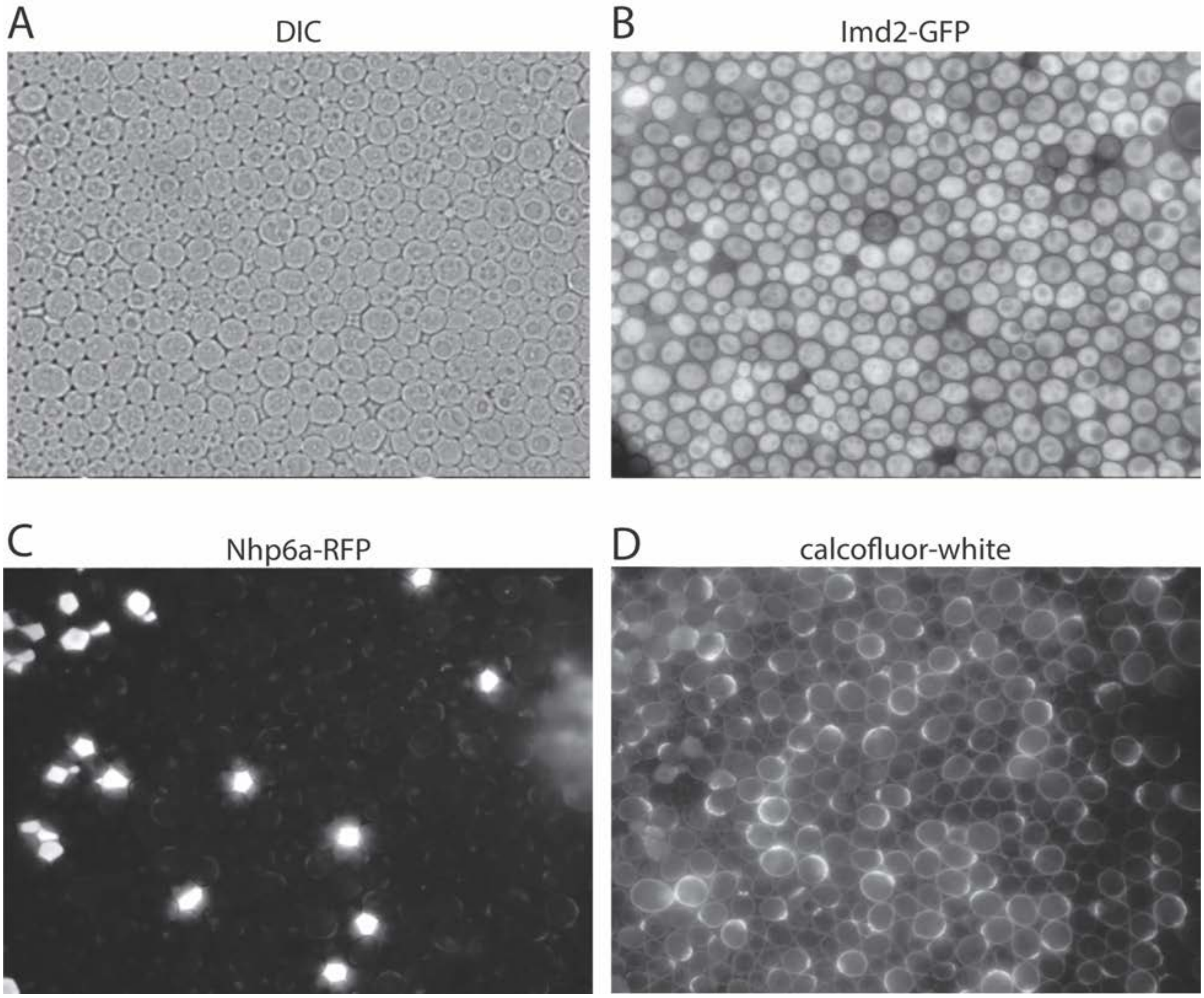

Supplemental Figure 4. Confluent cells after 50 hours of perfusion with 1.5 μg/mL MPA imaged with a 100x objective. (A) DIC. (B) Imd2-GFP. (C) Nhp6a-RFP. (D) Calcofluor-white staining of the cell wall. These micrographs are from the experiment shown in Figure 4B.

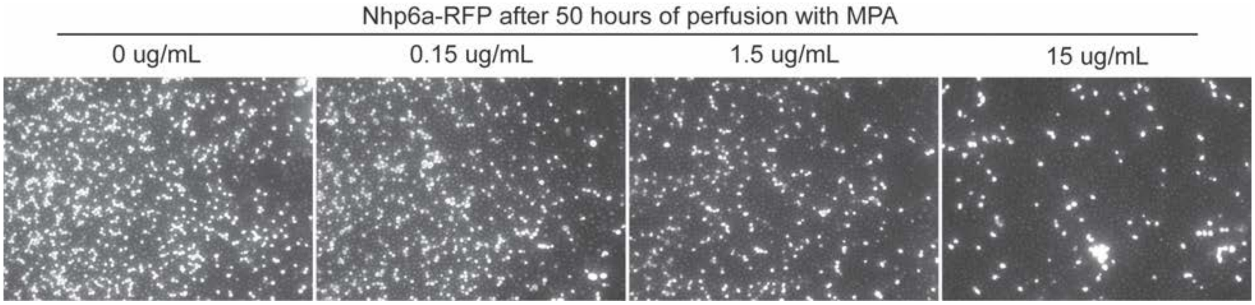

Supplemental Figure 5. Treatment with increasing MPA results in less accumulation of cells with brightly fluorescing Nhp6a-RFP. Nhp6a-RFP fluorescence after 50 hours of perfusion with medium containing the indicated concentration of MPA. These micrographs are from the experiment shown in Figure 3.

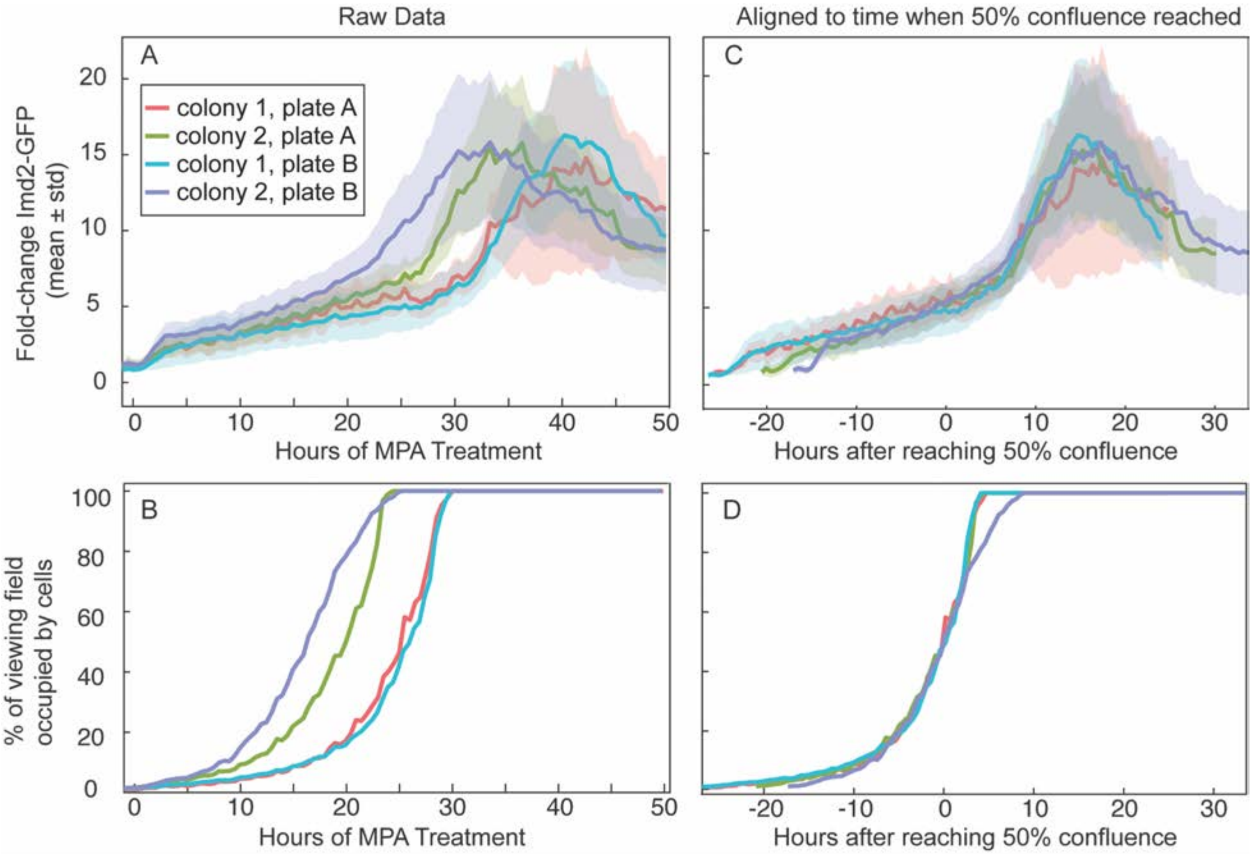

Supplemental Figure 6. Reproducibility of Imd2-GFP induction during 1.5 μg/mL MPA treatment in four biological replicates. (A) The population mean Imd2-GFP expression (solid lines) with one standard deviation above and below the mean (shaded areas) for four individual colonies picked from two plates streaked out on different days from the same frozen stock of cells. (B) The percentage of the viewing field occupied by cells as a function of time. The variable time required for each viewing field to reach maximum cell density was likely due to variability in the starting number of cells loaded into the viewing field (15-21) and the number of newly budded cells (0-3). (C) Imd2-GFP expression aligned by the time when cells reached 50% confluency. (D) Cell growth rate for each viewing field is approximately equivalent when aligned using the time when each field reached 50% confluency.

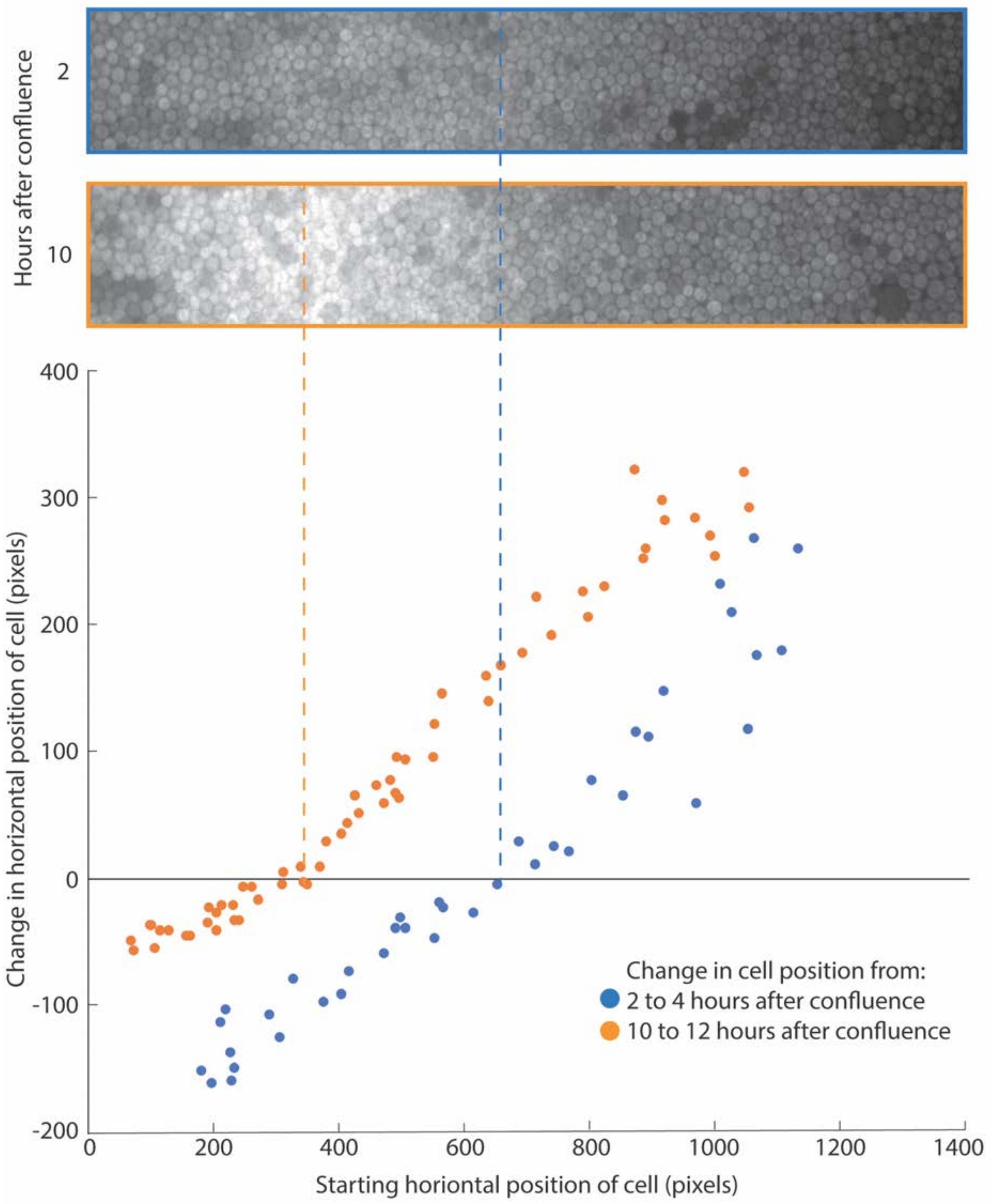

Hours after confluence
2
10
400
300
200
100
0
-100
-200
0
200
400
600
800
1000
1200
1400
Change in horizontal position of cell (pixels)
Starting horiontal position of cell (pixels)
Change in cell position from:
2 to 4 hours after confluence
10 to 12 hours after confluence

Supplemental Figure 7. The zone of maximum colony expansion shifts leftward away from the nutrient source over time. *Upper panels.* The central 20% of the viewing field (vertically) at either 2 (blue border) or 10 hours (orange border) after confluence for the Imd2-GFP channel of cells treated with 1.5 ug/mL MPA. The scale of each image corresponds to the horizontal axis of the graph shown below. *Lower panel.* The horizontal position of individual cells at 2 hours after confluence (blue) or 10 hours after confluence (orange) is plotted on the x-axis and their horizontal movement over the next two hours is plotted on the y axis. A negative number indicates movement to the left (away from the nutrient source) and a positive number indicates movement to the right. Dotted lines indicate the horizontal position of cells with no movement and where those cells are located within the Imd2-GFP viewing field shown in the still images above. Corresponds to data shown in Figure 4A and Figure 5.

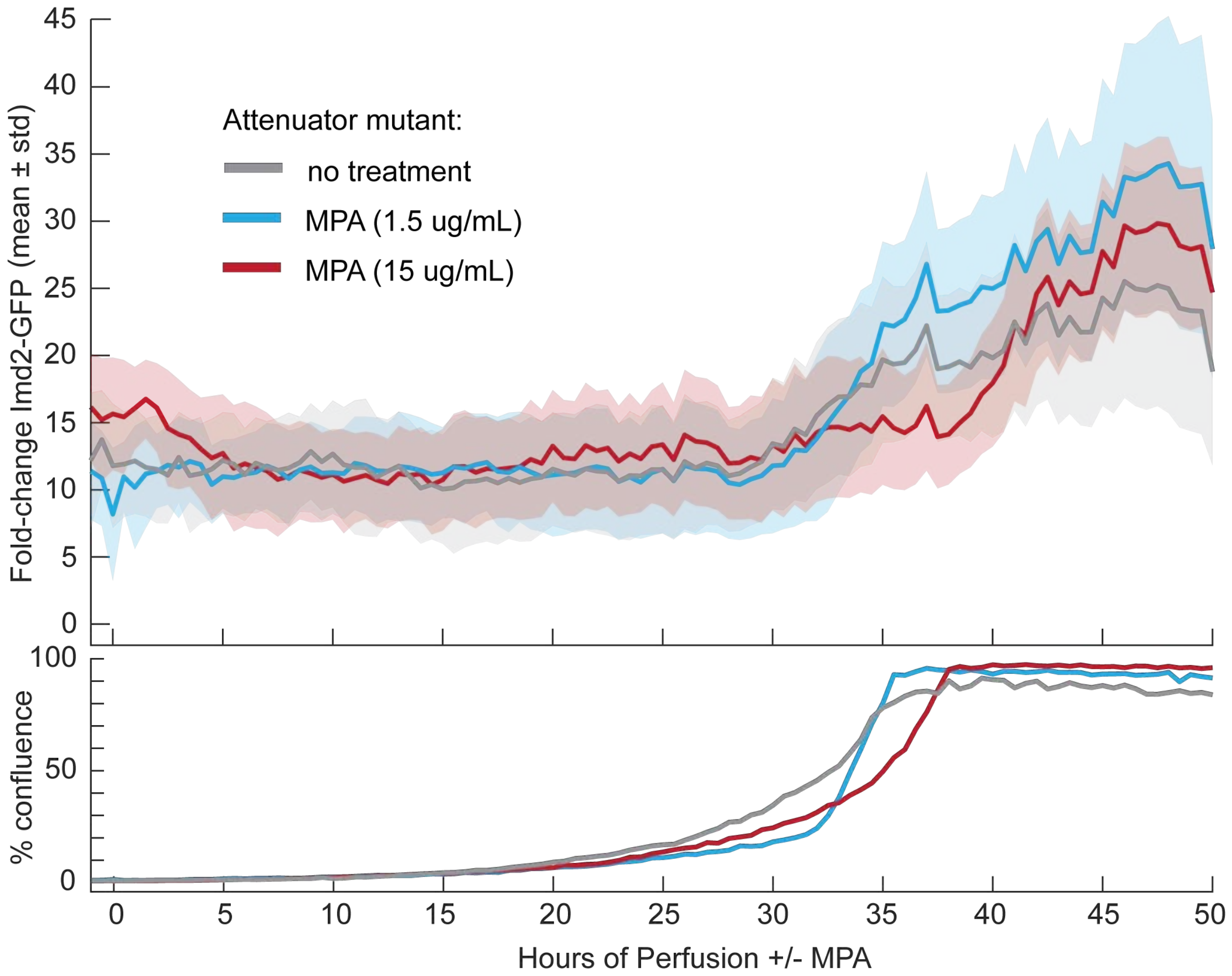

Supplemental Figure 8. The absence of the G start sites eliminates MPA induction of Imd2-GFP levels. *Upper panel.* Population mean Imd2-GFP fold-induction (solid lines) with one standard deviation above and below the mean (shaded area) for attenuator mutant (ELS107) cells perfused with no treatment (gray), 1.5 μg/mL MPA (blue), or 15 μg/mL MPA (red). *Lower panel.* The percentage of the viewing field occupied by cells over time.

Supplemental Table 1. Oligonucleotides used

Oligo Number	Oligo Name	Sequence (5' to 3')
1306	IMD2 C331A QC-F	GGG AAC TGG CTC TAT TGC TAT TAC CCA AGA AG
1307	IMD2 C331A QC-R	CTT CTT GGG TAA TAG CAA TAG AGC CAG TTC CC
1476	IMD2-GFP-downstream-rev	CTC CAG TGA AAA GTT CTT CTC C
1477	IMD2-bp1-bp18-fw	ATG GCC GCC ATT AGA GAC
1536	pGAC24-empty-oligo1	CGT TTT GGC TAC CTG TTA CTC G
1537	pGAC24-empty-oligo2	TCG ACG AGT AAC AGG TAG CCA AAA CGA GCT
1558	IMD2-SalI-rev	TTT TTG TCG ACT CAG TTA TGT AAA CGC
1559	IMD2-SacI-fw	TTT TTG AGC TCA TGG CCG CCA TTA GAG
1568	pGAC24-IMD2-StopCodon-QC-fw	GCG TTT ACA TAA CTG AGT CGA CCT AGA TAA G
1569	pGAC24-IMD2-StopCodon-QC-rev	CTT ATC TAG GTC GAC TCA GTT ATG TAA ACG C
1589	Pho12-StopCodon-fw	TAAATAGACCTAATATGATTTATG
1642	86fw-top	AACATTTAACCGGAGAATCTGTTTT
1643	86fw-bottom	AGATTCTCCGGTTAAATGTTGATCA
1644	IMD2-promoter-fw-SacI	TTTTTGAGCTCCCAAGTTGGAACAACAAACACAG
1645	IMD2-395bp-rev-SpeI	TTTTTACTAGTCTTAGCTTCACCAACGGTCG
1646	IMD2-deltaG-inversePCR-fw	ACC TTT TTT TCC GTA TTC TAT TCT ATT CC
1647	IMD2-deltaG-inversePCR-rev-EcoRI	TTC CAG CCG AAT TCT TTA CCA AAT ATC
